# Regulation of Cell Death Induced by Acetic Acid in Yeasts

**DOI:** 10.3389/fcell.2021.642375

**Published:** 2021-06-24

**Authors:** Susana R. Chaves, António Rego, Vítor M. Martins, Cátia Santos-Pereira, Maria João Sousa, Manuela Côrte-Real

**Affiliations:** ^1^Centre of Biological and Environmental Biology (CBMA), Department of Biology, University of Minho, Braga, Portugal; ^2^Centre of Biological Engineering (CEB), Department of Biological Engineering, University of Minho, Braga, Portugal

**Keywords:** acetic acid, apoptosis, biotechnology, mitochondria, regulated cell death, signaling pathways, vacuole

## Abstract

Acetic acid has long been considered a molecule of great interest in the yeast research field. It is mostly recognized as a by-product of alcoholic fermentation or as a product of the metabolism of acetic and lactic acid bacteria, as well as of lignocellulosic biomass pretreatment. High acetic acid levels are commonly associated with arrested fermentations or with utilization as vinegar in the food industry. Due to its obvious interest to industrial processes, research on the mechanisms underlying the impact of acetic acid in yeast cells has been increasing. In the past twenty years, a plethora of studies have addressed the intricate cascade of molecular events involved in cell death induced by acetic acid, which is now considered a model in the yeast regulated cell death field. As such, understanding how acetic acid modulates cellular functions brought about important knowledge on modulable targets not only in biotechnology but also in biomedicine. Here, we performed a comprehensive literature review to compile information from published studies performed with lethal concentrations of acetic acid, which shed light on regulated cell death mechanisms. We present an historical retrospective of research on this topic, first providing an overview of the cell death process induced by acetic acid, including functional and structural alterations, followed by an in-depth description of its pharmacological and genetic regulation. As the mechanistic understanding of regulated cell death is crucial both to design improved biomedical strategies and to develop more robust and resilient yeast strains for industrial applications, acetic acid-induced cell death remains a fruitful and open field of study.

## Introduction

Acetic acid, or ethanoic acid (CH_3_COOH), is a weak organic acid best known as a frequent by-product of the alcoholic fermentation carried out by *Saccharomyces cerevisiae* and the main component of volatile acidity in wine. Usually, alcoholic fermentation by *S. cerevisiae* in grape must yields low concentrations of acetic acid ranging from 0.1–0.3 g/L (1.7–5 mM) (Zamora, 2009). However, contaminating lactic and acetic acid bacteria can account for higher production of this acid, which may lead to undesirable threats to the fermentation process. Acetic acid concentrations above 0.9 g/L (15 mM) can noticeably alter wine organoleptic properties, but concentrations above 0.6 g/L (10 mM) can already negatively affect yeast fermentative performance (Ribéreau-Gayon et al., 2006; Sousa et al., 2012).

Apart from the traditional use of yeast in the production of alcoholic beverages, yeasts are also used in different biotechnological applications, such as in the production of bioethanol or of other commodities. As environmental concerns have motivated the search for efficient and less polluting methods to produce bio-based chemicals (including fuels), lignocellulosic biomass from different industrial residues and wastes arose as a good candidate for the so-called second generation biorefineries. However, yeasts do not assimilate the abundant sugar polymers present in these substrates, which have to be hydrolyzed to release simple fermentable sugars. Depending on feedstock and pretreatment conditions, high amounts of acetic acid can be released, reaching up to 11.5 g/L (191 mM) (Taherzadeh et al., 1997). For these acetic acid levels, cell death already occurs, which will negatively affect fermentation or even prevent it. On the other hand, the toxic effects of acetic acid can also be beneficial. For instance, since it can be cytotoxic to yeast and several other fungi, acetic acid has been explored along with other weak organic acids in the food industry as a natural preservative. Moreover, together with other short chain fatty acids, acetate is a product of the metabolism of acetic and propionic acid bacteria in the human gut. Though this is not the focus of the present review, acetate preferentially induces apoptosis in colorectal cancer (CRC) cells in comparison with non-cancer cell lines (Hague et al., 1995; Scheppach et al., 1995; Heerdt et al., 1997; Marchetti et al., 1997). The cytotoxicity of acetic acid and acetate in yeast and cancer cells is therefore of considerable interest due to its impact on the development of not only biotechnological but also biomedical applications.

The present review aims to cover the current understanding of cell death induced by acetic acid and how this knowledge paves the way to its modulation in yeast-based bioprocesses. For this purpose, we performed an extensive literature search on the Pubmed database with the keywords “yeast” and “acetic acid” and compiled information from published studies performed with concentrations of acetic acid that are lethal to wild-type controls and that shed light on regulated cell death mechanisms induced by this stimulus. For this reason, we manually eliminated reports that used sub-lethal concentrations of acetic acid, and therefore studies focusing on acetic acid tolerance (either on plates or in liquid media) are sometimes mentioned but are not the focus of this review. As such, when discussing the effects of acetic acid throughout the text, transient exposure to lethal concentrations is implied unless otherwise noted.

## Hallmarks of Acetic Acid-Induced Regulated Cell Death

Since the mid-80s, several studies showed that acetic acid is transported to the intracellular milieu of several yeast species, including *S. cerevisiae*, in its undissociated form by simple diffusion, and is not metabolized by cells grown in glucose-containing media. Since acetic acid has a pKa of 4.76 and the intracellular physiological pH is higher than pH values of commonly used media, acetic acid dissociates upon entering the cell and accumulates as a function of the ΔpH (Leão and Van Uden, 1986; Cássio et al., 1987). In addition, yeast cells can uptake this acid by active/mediated transport when grown under glucose derepression conditions, such as with lactic or acetic acid as carbon sources, or in glucose medium in species where acetic acid utilization is not under glucose repression (Casal et al., 1996; Sousa et al., 1996). The first reports on the cytotoxic effect of acetic acid toward yeast cells date back to 1989, when two types of cell death were ascribed to this monocarboxylic acid. Indeed, when specific death rates induced by 0–2% (w/v) (0–320 mM) acetic acid at pH 3.3 in *S. cerevisiae* were plotted as a function of the reciprocal absolute temperature (Arrhenius plots), two sub-groups of parallel straight lines with different slopes became apparent. This indicated that acetic acid induces two types of death: a high enthalpy death (HED, slope of 12.4 cal/mol), similar to that of thermal death, which predominates at high temperatures (37–40°C) and low acetic acid concentrations (<80 mM), and a low enthalpy death (LED, slope 3.9 cal/mol), which predominates at lower temperatures (20–26°C) and higher acetic acid concentrations (Pinto et al., 1989). This means that the effect of small temperature variations on cell death induced by acetic acid was higher at higher temperatures than at lower temperatures. It had already been established that the thermodynamic parameters of cell death activation could shed light on the nature and localization of the targeted sites of sub-products of alcoholic fermentation (Van Uden, 1984). At that time, LED was considered a consequence of cytoplasm acidification (Cardoso and Leão, 1992) and, as there was evidence indicating the inner mitochondrial membrane (IMM) could be a putative target in thermal death (Simões-Mendes et al., 1978), it was hypothesized that mitochondria could contribute to acetic acid-induced HED. However, further studies on acetic acid-induced cell death and on the involvement of mitochondria in a regulated process were only reported several years later.

It was first demonstrated that exposure of *S. cerevisiae* and of the non-conventional spoilage yeast *Zygosaccharomyces parabailii* ISA 1307 (formerly *Z. bailii*) to high concentrations of acetic acid (1–3% (v/v), 173–520 mM), pH 3.0, resulted in a late loss of plasma membrane integrity in both strains preceded by a deficit in vacuolar processing of the FUN-1 stain, reflecting a decrease in metabolic activity (Prudêncio et al., 1998). However, it was only in the beginning of the 21^*st*^ century that biochemical markers of cell death induced by acetic acid were characterized for the first time. Ludovico et al. (2001) showed that exposure of exponentially growing *S. cerevisiae* cells to low doses of acetic acid (20–80 mM) at pH 3.0 induced cell death associated with typical mammalian apoptotic markers, including chromatin condensation [by transmission electron microscopy (TEM)], phosphatidylserine (PS) exposure [by annexin V/propidium iodide (PI) staining] and DNA strand breaks [by terminal deoxynucleotidyl transferase dUTP nick end labeling (TUNEL) assay]. At the time, the process was defined as programmed cell death (PCD) (Ludovico et al., 2001), whereas nowadays it is designated as regulated cell death (RCD). Indeed, RCD is currently defined as a type of cell demise that occurs upon a mild stress and that can be pharmacologically or genetically modulated, while PCD is considered a type of RCD which occurs under physiological scenarios (Carmona-Gutierrez et al., 2018). On the other hand, under the same experimental conditions, high concentrations of acetic acid (120–200 mM) induced a necrotic phenotype, as suggested by extensive intracellular disorganization and compromised plasma membrane integrity (Ludovico et al., 2001). One year later, it became increasingly clear that an apoptotic-like cell death process can occur in yeast cells exposed to acetic acid, since two additional apoptotic markers, namely cytochrome *c* release from the mitochondria to the cytosol and moderate mitochondrial reactive oxygen species (ROS) accumulation, were identified. These events were associated with a decrease in oxygen consumption, mitochondrial transmembrane potential, cytochromes *a* + *a*_3_ levels and cytochrome *c* oxidase (COX) activity, highlighting the involvement of mitochondria in acetic acid-induced regulated cell death (AA-RCD), as discussed in the following sections (Ludovico et al., 2002). Mitochondrial structural and functional alterations were soon after reported for the acetic acid-resistant yeast *Z. parabailii* ISA 1307. In this yeast, treatment with up to 800 mM acetic acid was accompanied by mitochondrial structural changes, including swelling and reduction of cristae number, though the integrity of plasma and mitochondrial membranes were still preserved (Ludovico et al., 2003).

**FIGURE 1:**
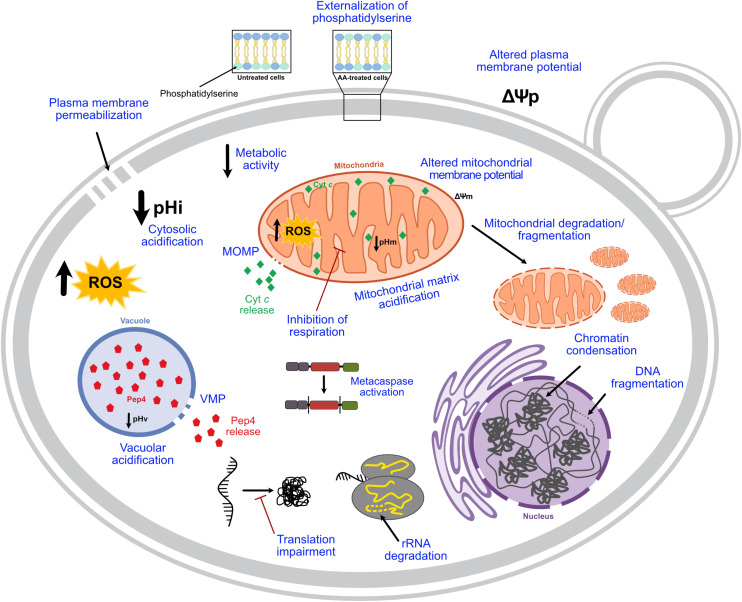
Current knowledge on the hallmarks of AA-RCD. When yeast cells are treated with lethal concentrations of acetic acid, a regulated cell death process characterized by typical mammalian apoptotic markers, highlighted in blue, can be triggered depending on the concentration. pHi, intracellular pH; pHv, vacuolar pH; pHm, mitochondrial pH; VMP, vacuolar membrane permeabilization; MOMP, mitochondrial outer membrane permeabilization; Cyt *c*, cytochrome *c*; Ψp, plasma membrane potential; Ψm, mitochondrial membrane potential.

AA-RCD hallmarks identified in early studies were further confirmed by several authors in the following years, and new features were described (Figure 1). ROS involvement in this process was characterized in more detail, and these species are currently considered mediators of AA-RCD (e.g., Giannattasio et al., 2005; Guaragnella et al., 2007). It was also reported that acetic acid increases caspase-like protease activity, as ascertained using fluorogenic and fluorescent caspase substrates, both in *S. cerevisiae* (Guaragnella et al., 2006, 2010a,b, 2011; Pereira et al., 2007), and in different *Candida* species (Aerts et al., 2009; Lastauskienë et al., 2014). In *S. cerevisiae*, caspase-like activity was highly dependent on the culture growth phase (Pereira et al., 2007), and was probably a consequence and not a cause of the cell death process, as more thoroughly discussed below. Acetic acid-induced mitochondrial degradation was demonstrated by several authors (Fannjiang et al., 2004; Pereira et al., 2010; Rego et al., 2012; Pereira et al., 2013; Dong et al., 2017; Martins et al., 2019) and it was shown that the normal tubular mitochondrial network becomes fragmented and deformed, acquiring a punctate pattern (Fannjiang et al., 2004; Pereira et al., 2010; Rego et al., 2012, 2018; Longo et al., 2015; Trindade et al., 2016), or even forming clusters in some mutant cells (Pereira et al., 2010; Rego et al., 2012) in an actin-dependent process (Pereira et al., 2010). Mitochondrial outer membrane permeabilization (MOMP) associated with cytochrome *c* release, a central event in mammalian intrinsic apoptosis, was also characterized: this hallmark in AA-RCD has been studied by assessing permeabilization of the outer mitochondrial membrane and maintenance of IMM integrity by *in vitro* enzymatic assays (Pereira et al., 2007; Rego et al., 2012, 2014a), and cytochrome *c* release by western blot and/or spectra analysis (Ludovico et al., 2002; Pereira et al., 2007; Giannattasio et al., 2008; Guaragnella et al., 2010a,b, 2011, 2013; Rego et al., 2014a, 2018, 2020; Guerreiro et al., 2016; Trindade et al., 2016; Martins et al., 2019). Besides mitochondria, a functional vacuole also seems to be crucial in AA-RCD (Schauer et al., 2009), involving vacuolar membrane permeabilization and subsequent release of Pep4p, the yeast ortholog of mammalian cathepsin D (CatD), which acts on mitochondrial degradation (Pereira et al., 2010). Acetic acid-induced DNA fragmentation was further characterized as single-strand DNA breaks and large DNA fragments, with no blunt-ended DNA double-strand breaks being identified (Ribeiro et al., 2006). Degradation of ribosomal RNA (rRNA) was also described by Mroczek and Kufel (2008), who demonstrated that acetic acid induces degradation of rRNA subunits 25S and 5.8S, though this phenotype was less evident in respiring cells owing to their higher oxidative stress defenses. Notably, rRNA degradation occurred before chromosomal DNA fragmentation (Mroczek and Kufel, 2008). Accordingly, acetic acid treatment leads to global impairment of translation associated with decreased levels of proteins involved in both initiation and elongation of translation (Almeida et al., 2009; Silva et al., 2013). One report indicated that acetic acid also slightly increases the levels of carbonylated proteins (Semchyshyn et al., 2011), though another found that overall protein carbonylation levels in acetic acid-treated cells are similar to those of untreated cells, but with distinct carbonylation patterns (Rego et al., 2012). Indeed, acetic acid-treated cells exhibited an enrichment in the carbonylation of some proteins, while others greatly decreased or disappeared (Rego et al., 2012). More recently, AA-RCD was associated with severe acidification of both the cytosol and mitochondrial matrix, although it is not clear what is the relative contribution of cellular accumulation of acetate and/or acidification to acetic acid cytotoxic activity (Dong et al., 2017). The same study showed additional ultrastructural changes such as cytosolic vacuolization, inhibition of lipid droplet accumulation and a smoother cellular surface. It has also been reported that acetic acid perturbs sphingolipid balance, which in turn regulates AA-RCD, by altering the mitochondrial levels of specific sphingolipid species, namely by decreasing most phosphorylated dihydro- and phytosphingosines and increasing the levels of dihydroceramide (Rego et al., 2012, 2018). Finally, acetic acid was also shown to impact mitochondrial phospholipids, inducing a reduction of almost 50% in their relative phosphatidylinositol content (Martins et al., 2019).

Supplementary Table 1 systematizes the aforementioned hallmarks employed by different authors to characterize AA-RCD in *S. cerevisiae* and also in other yeast species. Though a wide variety of structural and functional markers were used, it is apparent that most studies used several RCD markers as recommended by Carmona-Gutierrez et al. (2018), with assessment of ROS accumulation, plasma membrane integrity, PS exposure and DNA condensation and fragmentation as the most commonly used. The combined use of more than one marker is imperative to overcome not only methodological and technical limitations, for instance subsequent questions regarding the suitability of particular protocols and/or probes (Váchová and Palková, 2007; Kalyanaraman et al., 2012), but also lack of specificity to a given RCD subroutine and difficulties in discriminating RCD from other cellular processes.

In summary, the above-mentioned studies strongly support that exposure to acetic acid induces an RCD process associated with cell death markers typical of mammalian apoptosis. However, chemical or genetic modulation of cell death is the main feature that supports a regulated nature of a cell death process. As discussed in more detail in the next sections, several chemical compounds were found to modulate acetic acid-induced cell death and different gene products have been identified as anti- or pro-death factors. The term AA-RCD will be used throughout this review, but only when cell death markers confirming the process is regulated are shown. As such, cell death determined from data stemming only from cell viability assays, assessed by counting of colony forming units (CFUs) or spot assays after transient exposure, will be referred to as acetic acid-induced cell death (AA-CD).

## Chemical Modulation of Acetic Acid-Induced Regulated Cell Death

The effect of chemical compounds, with different mechanisms of action, on AA-RCD was assessed (Table 1) both to validate its controlled nature and to further characterize the associated cellular processes and dysfunctions.

**TABLE 1:** Overview of the effect of chemical compounds on AA-RCD.

Compound	Function	Survival phenotype	Strain	Associated hallmarks	Method	Reference
Cycloheximide	Protein synthesis inhibitor	Protection	*Sc* W303-1A	↓ Chromatin condensation	TEM	Ludovico et al., 2001
			*Sc* W303-1B	−	−	Guaragnella et al., 2006
			*Zp* ISA 1307	−	−	Ludovico et al., 2003
z-VAD-fmk	Pan-caspase inhibitor	No effect	*Sc* W303-1B	↓% FITC-VAD-fmk – stained cells	F-VAD/PI^FC^	Guaragnella et al., 2006
				↓ Cleavage of caspase substrates	Ac-FCS^SF^	Guaragnella et al., 2010a
MG132	Proteasome inhibitor	Slight protection	*Sc* W303-1B	↓ Chymotrypsin-, trypsin- and PGPH-like activities	Substrate hydrolysis^SF^	Valenti et al., 2008
Oligomycin	F_O_F_1_-ATPase inhibitor	No effect/slight sensitization	*Sc* W303-1A	−	−	Ludovico et al., 2002
Antimycin A	Inhibitor of complex III of electron transport chain	No effect	*Sc* W303-1B	Similar cytochrome *c* release	WB	Guaragnella et al., 2011
Oligomycin + Antimycin A	See single compounds	Sensitization	*Sc* W303-1B	−	−	Pereira et al., 2007
Cyclosporin A	Mitochondrial cyclophilin inhibitor	No effect	*Sc* W303-1B	−	−	Pereira et al., 2007
N-acetyl-L-cysteine (NAC)	Antioxidant	Protection	*Sc* W303-1B	↓ Cytochrome *c* release	WB	Guaragnella et al., 2010a
				↓ DNA fragmentation	TN/PI^FM^	
				↓ H_2_O_2_ accumulation	HFDA^FM^	
				↓ Cleavage of caspase substrates	Ac-FCS^SF^	

*The compounds as well as their function and induced phenotype toward AA-RCD, expressed by different cell death hallmarks, are presented. A summary of methods and results interpretations is listed as reported by the authors. Please refer to the individual manuscripts for details regarding protocols and controls. Ac-FCS, Ac-IETD/VEID-AMC, fluorogenic caspase-8/caspase-6 substrate; FC, flow cytometry; FM, fluorescence microscopy; F-VAD, FITC-VAD-fmk, fluorescein isothiocyanate conjugate of the pan-caspase inhibitor VAD(OMe)-FMK; HFDA, H_2_DCFDA, 2′,7′-dichlorodihydrofluorescein diacetate; PI, propidium iodide; SF, spectrofluorimeter; TN, terminal deoxynucleotidyl transferase dUTP nick end labeling (TUNEL); TEM, transmission electron microscopy; WB, western blot; Sc, Saccharomyces cerevisiae; Zp, Zygosaccharomyces parabailii.*

Several reports showed that inhibition of protein synthesis with cycloheximide protects cells from AA-RCD (Ludovico et al., 2001; Guaragnella et al., 2006, 2008), associated with a decrease in chromatin condensation as assessed by TEM (Ludovico et al., 2001). The enhanced survival conferred by cycloheximide was not restricted to *S. cerevisiae*, as it was also observed in *Z. parabailii* (Ludovico et al., 2003). These studies therefore supported AA-RCD as an active process, dependent on *de novo* protein synthesis.

In studies with higher eukaryotes, reversal of cell death by caspase inhibitors is often used to support the existence of apoptosis. However, the pan-caspase inhibitor z-VAD-fmk did not affect survival of either wild-type or metacaspase-deficient mutant cells, even though it slightly decreased the percentage of cells stained with FITC-VAD-fmk after exposure to acetic acid (Guaragnella et al., 2006), and decreased cleavage of the fluorogenic caspase substrates Ac-IETD-AMC and Ac-VEID-AMC (Guaragnella et al., 2010b). Indeed, while several reports indicate that AA-RCD is accompanied by increased proteolytic activity toward aspartic substrates (Guaragnella et al., 2006, 2010a,b, 2011; Pereira et al., 2007; Aerts et al., 2009; Lastauskienë et al., 2014), none have clearly demonstrated that increased proteolysis is required for cell death execution. Proteasomal chymotrypsin-, trypsin- and peptidylglutamylpeptide-like activity also slightly increased after exposure to acetic acid, but only a slight increase in viability was imparted by the proteasome inhibitor MG132 (Valenti et al., 2008). However, it is possible that increasing permeability of cells to MG132, which greatly enhances its effects (Liu et al., 2007), could have resulted in higher protection. On the other hand, it cannot be discarded that protection by this inhibitor may be due to increased stability of a given pro-survival factor and not to a decrease in overall proteolytic activity.

As mentioned above, ROS accumulation is one of the hallmarks of AA-RCD. Therefore, the use of antioxidants was also among the first attempts to modulate this process, but few studies have demonstrated robust protection from cell death using this strategy. Guaragnella et al. (2010b) analyzed the effects of N-acetyl-L-cysteine (NAC), a commonly used antioxidant, on several parameters after exposure of *S. cerevisiae* W303-1B cells to acetic acid. They found that NAC increased cell survival over time and reduced both cytochrome *c* release and TUNEL-positive cells at 150 min. NAC also decreased the percentage of DCF-positive cells after 15 min of exposure to acetic acid (Guaragnella et al., 2010b). In contrast, in mutants lacking cytochrome *c* or Yca1p, no differences were observed in cell viability or TUNEL staining despite a similar reduction in DCF-positive cells at 15 min (Guaragnella et al., 2010a; Antonacci et al., 2012). NAC also reduced proteolytic activity toward Ac-IETD-AMC and Ac-VEID-AMC in wild-type cells but not in the mutants (Guaragnella et al., 2010b), though acetic acid-induced proteolytic activity is likely unrelated to AA-RCD. Indeed, the same group later argued against a direct role of caspase-like activity in AA-RCD execution, since conditions were found where there was an increase in caspase-like activity but decreased AA-RCD (Guaragnella et al., 2011).

Since AA-RCD is a mitochondria-mediated process, multiple studies have addressed the effect of modulators of mitochondrial function in this process. Inhibition of oxidative phosphorylation with oligomycin, which inhibits the F_O_ portion of F_O_F_1_-ATP synthase, had no effect or slightly decreased survival of cells exposed to different concentrations of acetic acid (Ludovico et al., 2002). Later, Pereira et al. (2007) reported that oligomycin plus antimycin A (which inhibits ubiquinol-cytochrome *c* oxidoreductase (complex III) of the electron transport chain), increased AA-CD (Pereira et al., 2007). Another study showed that antimycin A by itself did not affect AA-RCD in wild-type cells (Guaragnella et al., 2011). However, all these studies were performed in fermentable media, where respiration is diminished. Nonetheless, yeast cells seem to be more sensitive to acetic acid in glucose-containing media than in non-fermentable or non-repressible carbon sources such as raffinose (Guaragnella et al., 2013). This is in agreement with a previous study showing that glucose-grown cells are more sensitive to acetic acid during exponential phase than in the stationary growth phase (Ludovico et al., 2002). Taken together, these results indicate that respiratory metabolism plays a protective role in AA-RCD, though other factors may be involved. On the other hand, a strain lacking mitochondrial DNA (mtDNA, ρ^0^) is resistant to acetic acid (Ludovico et al., 2002). Although these studies used opposite mating types of the W303 strain, this apparent contradiction may be due to a pleiotropic effect associated with the absence of mtDNA. Indeed, it was suggested that raffinose increases resistance to AA-RCD as a result of mitochondrial retrograde (RTG) pathway activation (Guaragnella et al., 2013), suggesting that activation of the RTG pathway in the absence of mtDNA can underlie the observed resistance of ρ^0^ cells (Parikh et al., 1987). In addition, there is evidence that repression of processes other than respiration by glucose can also underlie the higher sensitivity to AA-RCD observed in glucose-grown cells (Sousa et al., 2013), and this is a general feature in response to stress (Conrad et al., 2014).

It was also shown that cyclosporin A, which inhibits the mitochondrial cyclophilin, Cyp3p (Matouschek et al., 1995), consensually thought to regulate the opening of the permeability transition pore (PTP) in mammalian cells, does not significantly affect AA-RCD (Pereira et al., 2007).

Finally, Guerreiro et al. (2016) found that spermidine, a polyamine implicated in cell fate (Madeo et al., 2018), protects both *S. cerevisiae* and *Z. parabailii* ISA1307 from AA-CD, decreasing levels of ROS. As authors reported that acetic acid increases the levels of Spe3p, a protein involved in spermidine biosynthesis, in *Z. parabailii* but not in *S. cerevisiae*, they proposed that increased spermidine synthesis may underlie the high acetic acid tolerance of this *Z. parabailii* strain (Guerreiro et al., 2016).

## Genetic Modulation of Acetic Acid-Induced Regulated Cell Death

After the discovery that *S. cerevisiae* undergoes an active cell death program upon acetic acid challenge that resembles mammalian intrinsic apoptosis, it was only a matter of time before studies arose trying to pinpoint the molecular players within this process. Indeed, potential yeast orthologs of mammalian apoptosis regulators were investigated and phenotypically characterized through gene editing techniques, and a large number of genes were shown to play a role in AA-RCD. In this section, we present an overview of the twenty-year long research that has contributed to the elucidation of the intricate molecular machinery that regulates yeast AA-RCD, which is summarized in Supplementary Table 2.

### The Yeast Metacaspase

A major hallmark of mammalian apoptosis is the activation of caspases, cysteine proteases that are activated in cascade when apoptosis is triggered and mediate the dismemberment of cellular components (Van Opdenbosch and Lamkanfi, 2019). The yeast *S. cerevisiae* does not encode obvious caspase orthologs and the only known caspase-like family member is the metacaspase Yca1p (yeast caspase-1), which exhibits caspase-like proteolytic activity and can be induced upon apoptosis triggering (Madeo et al., 2002; Mazzoni and Falcone, 2008). The role of this metacaspase in AA-RCD has been explored in several studies. Fannjiang et al. (2004) showed that abrogation of Yca1p restores FUN-1 processing ability in acetic acid-challenged cells. Still, although *yca1*Δ cells are more resistant to acetic acid than the wild-type strain in terms of cell survival, multiple studies showed no significant difference in apoptotic markers such as chromatin condensation (Guaragnella et al., 2006), DNA fragmentation (Guaragnella et al., 2010a; Longo et al., 2015) or mitochondrial fragmentation (Longo et al., 2015), though Yca1p was required for cytochrome *c* to be released from mitochondrial reservoirs (Guaragnella et al., 2010a). In *S. pombe*, acetic acid treatment increased mRNA levels of pombe caspase-1 (*PCA1)* and acridine orange/ethidium bromide (AO/EtBr) staining, but authors showed only increased growth of *pca1*Δ cells on plates containing acetic acid, not protection from cell death (Agus et al., 2020). Of note, as discussed above, the caspase-like activity observed in acetic acid-treated cells (Pereira et al., 2007; Aerts et al., 2009; Guaragnella et al., 2010a,b, 2011; Lastauskienë et al., 2014; Saraiva et al., 2006) may be a consequence and not a direct mediator of the cell death process. In fact, neither a pan-caspase inhibitor is able to prevent AA-RCD (Guaragnella et al., 2006), nor, to our knowledge, have specific metacaspase substrates in AA-RCD been identified. Overall, these studies suggest that *YCA1* disruption *per se* does not fully abolish AA-RCD, suggesting the involvement of a metacaspase-independent pathway. Under that scenario, it is plausible that the protection from AA-RCD imparted by *YCA1* deletion is associated with a yet unidentified role of this protein in other cellular mechanisms. Indeed, several non-apoptotic roles have been attributed to Yca1p, and it has been suggested that *yca1*Δ cells may be inherently resistant to stress stimuli independently of a role in cell death (Lee et al., 2010; Longo et al., 2015; Ždralević et al., 2015; reviewed in Hill and Nyström, 2015).

### Mitochondrial Regulators

As mentioned previously, the involvement of mitochondria in yeast AA-RCD was first established by our laboratory long ago (Ludovico et al., 2002), and since then there has been a plethora of research directed toward identifying mitochondrial effectors in this type of cell death, as described below.

#### Mitochondrial Respiratory Complexes

Cytochrome *c* is a water-soluble intermembrane space protein that is a key player in both life and death decisions within the cell: it is responsible for the transfer of electrons from cytochrome *bc*_1_ reductase (complex III) to cytochrome *c* oxidase (complex IV) in the electron transport chain, as well as for the formation of the apoptosome during mammalian apoptosis (Hüttemann et al., 2011). As expected, several studies have dissected the role of cytochrome *c* in yeast AA-RCD. Although deletion of the cytochrome *c* isoforms *CYC1* and *CYC7* increased cell viability in comparison to the wild-type strain, it did not completely abolish cell death (Guaragnella et al., 2010a). As such, cytochrome *c* is not strictly required for AA-RCD to occur. Deletion of *CYC3*, coding for a cytochrome *c* heme lyase, which attaches heme to apo-cytochrome *c*, has also been associated with resistance to acetic acid, though only through clonogenic survival assays (Ludovico et al., 2002). Moreover, expression of a mutant form of Cyc1p that is unable to reduce cytochrome *c* oxidase (W65S substitution, ^*W65S*^Cyc1p), results in resistance to AA-RCD in comparison to control and *cyc1,7*Δ yeast cells (Guaragnella et al., 2011). Cells expressing ^*W65S*^Cyc1p exhibited increased survival along with reduced DNA fragmentation and ROS accumulation. No cytochrome *c* release was detected, though caspase-like protease activity was increased in comparison to wild-type cells. Thus, it seems that cytochrome *c* is able to modulate caspase-like activity despite not being released from mitochondria (Guaragnella et al., 2011). More recently, it was shown that expression of a permanently membrane-anchored cytochrome *c* recombinant protein (Cyc1^MA^) in a *cyc1,7*Δ background sensitized cells in the absence of cell death stimuli during exponential growth, which was corroborated by a diminished chronological lifespan (Toth et al., 2020). Cyc1^MA^ cells displayed a slight decrease in clonogenic survival and mitochondrial transmembrane potential, along with increased PI and dihydroethidium (DHE) staining, in comparison with both wild-type and *cyc7*Δ cells. These phenotypes were not due to respiratory deficiency, since cells expressing Cyc1^MA^ exhibited a normal assembly of mitochondrial respiratory supercomplexes and wild-type-like aerobic cell growth. Nevertheless, these phenotypic differences were exacerbated upon acetic acid treatment, with Cyc1^MA^ cells evidencing a much greater reduction in clonogenic survival along with increased PS exposure and ROS accumulation. Unsurprisingly, no cytochrome *c* was released from mitochondria (Toth et al., 2020). In summary, it is apparent that although cytochrome *c* release occurs during AA-RCD, it is not necessary for this process. Moreover, it has not been ascertained whether it plays a role in cytosol, but loose binding of cytochrome *c* to the IMM is required to maintain cellular homeostasis with or without the presence of a death stimuli.

Apart from cytochrome *c*, alterations in the expression of other respiratory complex proteins also affect sensitivity to acetic acid. For instance, abrogation of the β subunit of the F_1_ part of the F_O_F_1_-ATPase (complex V), encoded by *ATP2*, sensitizes cells to AA-CD (Pereira et al., 2007). On the other hand, Ludovico et al. (2002) showed that deletion of a gene coding for an assembly factor for the F_O_ part of the ATPase, *ATP10*, rendered cells more resistant to acetic acid. The authors further determined that a fully assembled mitochondrial F_O_F_1_-ATPase is required for translocation of cytochrome *c* from mitochondria to the cytosol, and that this phenotype is not due to impaired respiration, since oligomycin-treated wild-type cells did not display enhanced survival, as referred above (Ludovico et al., 2002).

#### Mitochondrial Fission/Fusion Machinery

Mitochondria are remarkably plastic organelles and form dynamic interconnecting networks where fusion mechanisms (mediated in yeast by Fzo1p and Mgm1p) promote neutralization of damaged mitochondrial components, while fission mechanisms (mediated in yeast by Dnm1p) allow the network to dispose of damaged mitochondria (Suen et al., 2008; Cosentino and García-Sáez, 2014). In 2004, Fannjiang et al. showed for the first time that acetic acid can induce mitochondrial fragmentation in yeast cells. The mitochondrial fission proteins Dnm1 and Mdv1 appeared to promote AA-CD, as *dnm1*Δ and *mdv1*Δ strains displayed a resistant phenotype as evidenced by increased clonogenic survival and FUN-1 processing ability in comparison with the wild-type strain (Fannjiang et al., 2004). On the other hand, ablation of a mitochondrial fission factor required for Dnm1p recruitment, Fis1p, resulted in sensitivity to this stressor, with a significant decrease in cell viability and an increase in PS exposure and chromatin condensation (Fannjiang et al., 2004; Sj et al., 2019). However, the same group found that the phenotypes encountered in acetic acid-challenged *fis1*Δ cells are a consequence of secondary mutations in *WHI2* (Cheng et al., 2008) and *SIN3* genes (Teng et al., 2013). Expression of wild-type Whi2p regulated by its endogenous promoter reverts the cell death phenotype, indicating that the sensitivity to acetic acid displayed by *fis1*Δ cells is due to *WHI2* genetic loss-of-function, which protects cells from mitochondrial respiratory defects (Cheng et al., 2008). Importantly, this mutation was not found in either *dnm1*Δ or *mdv1*Δ cells, indicating that *WHI2* loss-of-function compensates for an unknown role of Fis1p other than mitochondrial fission (Cheng et al., 2008). Similarly, the *SIN3* mutation induced by *FIS1* deletion is able to recover the defective growth of *fis1*Δ cells, but the role of this mutation in AA-RCD was not ascertained (Teng et al., 2013). Fis1p was not required for mitochondrial fragmentation upon acetic acid exposure, but the typical mitochondrial fragmentation observed during latrunculin A-induced actin disruption was absent in *fis1*Δ cells, indicating that the fission machinery is different under death stimuli and in healthy cells (Fannjiang et al., 2004). Of note, mitochondrial fragmentation by itself is not sufficient to induce cell death, as lack of the mitochondrial fusion factor Fzo1p does not exacerbate sensitivity to acetic acid even though *fzo1*Δ mitochondria are predominantly fragmented (Fannjiang et al., 2004).

#### Mitochondria-to-Nucleus Crosstalk

Apoptosis-inducing factor (AIF), as its name indicates, plays a central role in mammalian apoptotic cell death. In healthy cells, AIF is a membrane-bound protein confined to mitochondria, where it acts as an oxidoreductase. However, upon apoptotic stimuli, this protein translocates to the nucleus, where it promotes DNA fragmentation and chromatin condensation (Susin et al., 1999). It was reported that *S. cerevisiae* and *C. albicans* also possess an ortholog of AIF, Aif1p (Wissing et al., 2004; Ma et al., 2016). However, Amigoni et al. did not find differences in survival between wild-type and *S. cerevisiae aif1*Δ cells exposed to acetic acid, though deletion of *AIF1* protected a sensitive mutant strain, lacking Hxk2p, from AA-RCD (Amigoni et al., 2016). In *C. albicans*, deletion of *AIF* slightly decreased, whereas overexpression slightly increased, the percentage of Annexin V/PI-positive cells induced by low concentrations of acetic acid, as well as ROS levels, though no effect was observed for higher concentrations (Ma et al., 2016). Nonetheless, clonogenic survival assays were not performed and, therefore, a possible role for Aif1p in AA-RCD is still not fully characterized.

Nuc1p, the yeast homolog of endonuclease G, is a mitochondrial nuclease that, like mammalian Aif1p, is translocated to the nucleus upon apoptotic stimuli, where it degrades both DNA and RNA. Interestingly, both overexpression and abrogation of this protein sensitized yeast cells to acetic acid, indicating that Nuc1p displays both a protective and mediating role in this process (Büttner et al., 2007, 2011). The authors further demonstrated that a fully functional nuclease domain is required for cell death rescue (Büttner et al., 2007). Remarkably, deletion of *NUC1* decreased survival of yeast cells exposed to acetic acid in glucose media, accompanied by increased ROS accumulation, but conferred protection to cells cultured in glycerol-containing media. Cells lacking Nuc1p were found to die by necrosis on fermentative media but not on respirable media, meaning that Nuc1p mediates apoptotic-like cell death only when oxidative phosphorylation is not repressed (Büttner et al., 2007).

Apart from proteins that may be translocated to the nucleus, multiple studies have investigated the involvement of mitochondria-to-nucleus communication through the RTG-dependent retrograde signaling pathway in AA-RCD. For instance, cells lacking Rtg2p displayed decreased cell survival after exposure to acetic acid associated with increased DNA fragmentation, ROS accumulation and PS exposure, though with no noticeable increase in cytochrome *c* release (Guaragnella et al., 2013; Laera et al., 2016). Rtg2p is a positive regulator of the RTG pathway, as it inhibits Mks1p, thus allowing nuclear translocation of the Rtg1/3p transcription factors and induction of RTG target gene expression (Dilova et al., 2002; Komeili et al., 2000). Those results therefore indicate that activation of the retrograde pathway protects cells from AA-RCD when respiratory metabolism is de-repressed. However, Rtg2p may play other roles, as deletion of *RTG2* also sensitized yeast cells to AA-RCD under conditions where the retrograde response was not activated (Guaragnella et al., 2019). On the other hand, RTG pathway activation through abrogation of the negative regulator Mks1p (Sekito et al., 2002) had no effect on AA-RCD in media with standard 2% glucose, although partial resistance was observed with low glucose concentrations (Guaragnella et al., 2013). As this suggests that relief from carbon catabolite repression (CCR) is required for the observed resistance, authors further analyzed the contribution of Snf1p-dependent transcriptional activators that control expression of glucose-repressible genes. They found that Adr1p and Cat8p interact with Rtg2p and with each other to increase resistance to AA-RCD in raffinose media, but no role was found for Hap4p, the regulatory subunit of the Hap2,3,4,5 complex (Laera et al., 2016). Taken together, data suggest that both respiratory metabolism and central carbon metabolism reprogramming modulate AA-RCD.

#### The Endoplasmic Reticulum-Mitochondria Circuit

The unfolded protein response (UPR) is activated in response to misfolded proteins in the endoplasmic reticulum (ER), which in turn activate stress response mechanisms to maintain homeostasis. However, when this pathway fails to correctly refold proteins, apoptosis is induced (Wu et al., 2014). Exposure to acetic acid resulted in a 10-fold augmented expression of the ER molecular chaperone *KAR2*, which aggregates with misfolded proteins during ER stress, as well as in UPR activation through the Ire1p-Hac1p pathway (Kawazoe et al., 2017). Indeed, upon sensing misfolded proteins, Ire1p was activated by self-aggregation and promoted splicing of the transcription factor *HAC1* pre-mRNA, which in turn increased *KAR2* mRNA levels (Kawazoe et al., 2017). Deletion of *IRE1* or *HAC1* resulted in increased sensitivity to acetic acid, highlighting the importance of the ER stress response in maintaining cellular homeostasis in response to acetic acid exposure (Kawazoe et al., 2017). If failing to do so, apoptosis is induced through the mitochondrial pathway (Bhat et al., 2017).

A subsequent study showed that the Endoplasmic Reticulum-Mitochondria Encounter Structure (ERMES), a physical point of crosstalk between the ER and mitochondria, is involved in AA-RCD (Martins et al., 2019). Though ERMES is thought to participate in lipid exchange and calcium signaling, its role is not yet fully understood (Michel and Kornmann, 2012). This complex is formed by the ER-anchored protein Mmm1p, cytosolic Mdm12p and two outer mitochondrial membrane proteins, Mdm10p and Mdm34p. Martins et al. (2019) showed that *mdm10*Δ, *mdm12*Δ, and *mdm34*Δ cells display a resistant phenotype upon acetic acid challenge, exhibiting a significant delay in the appearance of several apoptotic markers such as loss of mitochondrial transmembrane potential, mitochondrial degradation and ROS accumulation, as well as in the loss of plasma membrane integrity evidencing secondary necrosis. Additionally, cells lacking either Mdm10p or Mdm34p were unable to release cytochrome *c* from mitochondria, while *mdm12*Δ cells lost the majority of their mitochondrial cytochrome *c* pool. These results hinted that the mitochondria-bound members of ERMES might modulate MOMP (Martins et al., 2019). In agreement with this hypothesis, cytochrome *c* release was also decreased in *mdm34*Δ yeast cells heterologously expressing a constitutively active form of the pro-apoptotic protein Bax (Légiot et al., 2019). On the other hand, phospholipid analysis revealed that the ERMES function in lipid exchange between the ER and mitochondria does not seem to play a role in the alterations of mitochondrial phospholipid content induced by acetic acid. Indeed, both wild-type and mutant cells displayed a similar reduction of almost 50% in their relative content of mitochondrial phosphatidylinositol after exposure to acetic acid. Nonetheless, differences in the mitochondrial phospholipidic profile observed between the strains lacking ERMES components and wild-type cells before treatment may underlie the observed acetic acid resistance phenotype of the mutants (Martins et al., 2019).

#### Other Mitochondrial Regulators

Pereira et al. (2007) assessed the role of mitochondrial proteins that were considered putative components of the mammalian PTP at the time. Authors found that abrogation of the Aac1/2/3 protein isoforms, yeast orthologs of the adenine nucleotide translocase, significantly delays AA-RCD. The *aac1/2/3*Δ strain revealed a later onset of common apoptotic features in comparison to the control strain, with the exception of ROS accumulation, a phenotype that was not due to impaired respiration (Pereira et al., 2007). This work also assessed the role of other presumed PTP components such as Por1p, the yeast voltage-dependent anion channel, and Cpr3p, the yeast mitochondrial cyclophilin. Whilst *POR1* deletion increased yeast sensitivity to acetic acid, suggesting that Por1p functions as a negative regulator of the RCD program, Cpr3p deletion did not affect cell survival, in agreement with a lack of effect of cyclosporin A in AA-RCD in wild-type cells (Pereira et al., 2007). Later, another study showed that the sensitivity displayed by *por1*Δ mutants is not observed in the absence of Aac1/2/3 proteins, suggesting a role for Por1p in the negative regulation of cell death through inhibition of the pro-death function of Aac1/2/3 proteins (Trindade et al., 2016).

Yeast cells do not encode true Bcl-2 family members, but a protein containing a putative BH3 domain (Ybh3p) has been found (Büttner et al., 2011). Ybh3p overexpression led to a Bcl-X_*L*_-reversible sensitization to acetic acid that was accompanied by increased ROS accumulation, DNA fragmentation, PS exposure and cytochrome *c* release, while deletion of *YBH3* reduced AA-RCD (Büttner et al., 2011). Overexpressed GFP-Ybh3p localized to the vacuole in untreated cells but was partially translocated to mitochondria upon acetic acid treatment, which coincided with mitochondrial cytochrome *c* release, independently of mitochondrial transmembrane potential (Büttner et al., 2011). Authors therefore proposed that Ybh3p functions similarly to human Bax, as they also demonstrated that Ybh3p-FLAG interacts with Bcl-X_*L*_. In addition, deletion of *UTH1*, which prevents mitochondrial effects of Bax expression in yeast (Camougrand et al., 2003), decreased cell death and ROS accumulation in response to Ybh3p-FLAG overexpression in acetic acid-treated cells. However, authors did find Ybh3p-FLAG in mitochondria of untreated cells by sub-cellular fractionation, which disappeared after exposure to acetic acid, indicating it was released or degraded (indeed, smaller FLAG-tagged fragments were detected). Of note, although a role in AA-RCD was not ascertained, this same protein had been described as anti-apoptotic in a different study (Cebulski et al., 2011). FLAG-tagged Ybh3p was found to interact with Mir1p, an IMM phosphate carrier, and Cor1p, a subunit of cytochrome *bc*_1_ complex, and authors suggested they function on the same pathway, as deletion of *MIR1* or *COR1* increased viability of cells overexpressing Ybh3p-FLAG exposed to acetic acid (Büttner et al., 2011). Taken together, these results indicate that Ybh3p plays a role in AA-RCD, though further studies are necessary to elucidate the mechanism involved.

### Genes Involved in Antioxidant Defenses

As exposure to acetic acid triggers ROS accumulation, the role of cellular antioxidant defenses in AA-RCD was addressed both by assessing the activity of catalase (CTT) or superoxide dismutase (SOD), and by deleting or overexpressing genes coding for the proteins involved. Nonetheless the results obtained by several groups do not provide a definitive answer. Indeed, one study reported that exposure to acetic acid does not affect CTT or SOD activities of W303-1B cells (Giannattasio et al., 2005). However, pre-adaptation to an acidic medium in YPD pH 3.0 for only 30 min was shown to increase CTT but not SOD activity, which was not further altered by acetic acid. Since this adaptation protected cells from AA-RCD, authors proposed that detoxification of hydrogen peroxide, and not superoxide, may have a major role in preventing yeast AA-RCD. Another study by the same group showed that SOD but not CTT activity increased with pH 3.0, and that acetic acid decreased CTT activity in both wild-type and cells overexpressing the cytosolic catalase T (*CTT1)* or cytosolic superoxide dismutase (*SOD1)* (Guaragnella et al., 2008). Recently, these authors reported that acid stress increases CTT activity and subsequent exposure to acetic acid slightly reduces it, although to levels still higher than in untreated cells (Guaragnella et al., 2019). Yet, another study by Semchyshyn et al. reported that a shift to acidic media slightly increased SOD but not CTT activity in the same background. However, in that case, acetic acid increased both CTT and SOD activities. In another background (YPH250 strain), acetic acid increased the activity of SOD and CTT, though acidic media did not display a noticeable effect in this case. Deletion of the transcription factor *YAP1* in the YPH250 strain decreased SOD activity and prevented the previously observed acetic acid-induced SOD activity increase, while CTT activity slightly increased similarly to the wild-type strain. Moreover, in the same background, deletion of both genes encoding *S. cerevisiae CTT* had no effect on AA-RCD, whereas deletion of both *SOD* genes slightly increased sensitivity to acetic acid but only to 200 mM, as did the deletion of *YAP1* (Semchyshyn et al., 2011). In all, conditions reported in these studies were dissimilar, both in growth phase, media composition, and use of plasmid-bearing strains. Therefore, it is difficult to state how acetic acid affects SOD and CTT activities, though there is a tendency to observe that increased CTT activity protects cells from AA-RCD. In agreement, overexpression of *CTT1* from a *GAL* inducible promoter increased CTT activity, and resulted in resistance to AA-RCD. However, unexpectedly, the same study showed that overexpression of *SOD1* greatly increased sensitivity to acetic acid, even though there was a slight increase in SOD activity. This higher sensitivity was related with decreased CTT activity observed in *SOD1*-overexpressing cells (Guaragnella et al., 2008).

### Nuclear Regulators

DNA modification through histone acetylation has been suggested to play a role in AA-RCD. Dong et al. reported up-regulation of genes involved in histone acetylation and down-regulation of genes involved in histone deacetylation, and suggested that exposure to acetic acid increases histone acetylation. However, they showed a slight increase in PI-positive cells induced by acetic acid (150 mM) both when deleting or overexpressing several of the genes involved, which indicates that histone acetylation imbalance sensitizes cells to AA-RCD. More specifically, overexpression of *ADA2*, *AHC2*, *ESA1*, *EPL1*, *YAF9*, and *SET2*, as well as deletion of *ADA2*, *AHC2*, *HPA2*, *HOS1*, *SGF29*, and *YAF9*, increased the number of cells stained with Annexin V/PI and PI in response to acetic acid, whereas overexpression of *HPA2* decreased it. They further pre-treated cells with the histone deacetylase inhibitor sodium butyrate and found that concentrations over 20 mM enhanced the percentage of PI-stained cells in response to acetic acid. However, histone acetylation levels were not assessed (Dong et al., 2017). On the other hand, Vall-llaura et al. proposed that mutations in cysteines of the NAD^+^ dependent histone deacetylase *SIR2* lead to decreased Sir2p-dependent Pck1p deacetylation and associated metabolic changes, which resulted in increased growth in the presence of acetic acid (Vall-Llaura et al., 2019). Accordingly, it was shown that individual deletion of any of the genes coding for the components of the Set3 histone deacetylase complex (*CPR1*, *HOS2*, *HOS4*, *HST1*, *SET3*, *SIF2*, *SNT1*) decreases AA-CD, pointing to a protective role of acetylation in AA-RCD, though in this case cell death parameters of specific mutants were not analyzed (Sousa et al., 2013).

Tel1p (telomere maintenance 1) is a protein kinase involved in the cell cycle checkpoint response to DNA double-strand breaks by phosphorylating the H2A histone that is critical for cell cycle arrest during G1/S transition and also for telomere length regulation. Indeed, *tel1*Δ cells have 7-fold more shortened telomeres than wild-type cells (Di Domenico et al., 2014). Cells lacking Tel1p appear to be more sensitive to acetic acid and display increased PI-staining in comparison to the wild-type strain, which could be rescued by incubation with the anti-apoptotic natural compound quercetin. Although acetic acid-treated *tel1*Δ cells showed about 20% more cells classified as apoptotic by AO/EtBr staining than wild-type cells, additional quantitative assays are required to better characterize the *tel1*Δ phenotype regarding AA-RCD (Alugoju et al., 2018). Nevertheless, these data point to a possible involvement of cell cycle and telomere length in AA-RCD that could be further explored. Lastly, cells lacking Nma111p, a member of the mammalian apoptotic Omi/HtrA2 family, which in yeast is nuclear and not mitochondrial, also displayed resistance upon acetic acid challenge. However, this phenotype was only assessed through clonogenic survival assays and no studies addressed its localization after acetic acid exposure (Sokolov et al., 2006).

### Vacuolar Regulators

Multiple evidence demonstrates that acetic acid perturbs the vacuole and its associated functions, which in turn seems to be directly related with its effect on pH homeostasis. Indeed, acetic acid induces vacuolar and cytosolic acidification in different physiological scenarios (Carmelo et al., 1997; Schauer et al., 2009; Dong et al., 2017), with this latter alteration considered one of the key events leading to AA-RCD (Dong et al., 2017). As the proton pump Pma1p closely cooperates with the vacuolar proton pump V-ATPase to maintain pH homeostasis (Deprez et al., 2018), one could hypothesize that cells try to revert acetic acid-induced intracellular acidification by promoting extrusion of hydrogen ions from the cytosol to the extracellular space (by increasing Pma1p activity) or to the vacuole (leading to vacuolar acidification likely mediated by increased V-ATPase activity). Regarding the former, although a role of Pma1p in AA-RCD has not been addressed, Pma1p activation by sub-lethal concentrations of acetic acid does not counteract either the cytosolic or the vacuolar pH drop (Carmelo et al., 1997). In agreement with the later hypothesis, deletion of different V-ATPase genes (*VMA1-8*, *VMA13* and *VMA16*) sensitizes cells to this stressor, as shown in genome-wide screens aiming to identify players involved either in acetic acid tolerance (Kawahata et al., 2006; Mira et al., 2010) or AA-RCD (Sousa et al., 2013), reinforcing the role of this proton pump in coping with the pH perturbations induced by acetic acid.

A different study showed that yeast strains lacking genes coding for Class C Vacuolar Protein Sorting (VPS) proteins (*pep3*Δ, *pep5*Δ, *vps16*Δ, and *vps33*Δ), which lack an identifiable vacuole, are especially sensitive to acetic acid (Schauer et al., 2009). Exposure of these mutants to acetic acid triggered massive necrotic cell death at pH 3.0, though not at pH 4.05. Intracellular pH of cells exposed to acetic acid at the lower pH, but not at the latter, did decrease more in the mutants than in the wild-type strain, which could account for the phenotype. However, decreasing the intracellular pH of wild-type cells to levels comparable to the mutants by increasing the acetic acid concentration at pH 3.0 resulted in similar levels of cell death, though it did not lead to massive necrosis as monitored by PI staining. These findings were restricted to the Class C VPS mutants, as they were not observed in mutants lacking other vacuolar proteins (*vma6*Δ, *vma8*Δ, *vps19*Δ, or *vma13*Δ). Nonetheless, the authors concluded that a functional vacuole is necessary for AA-RCD, though not for hydrogen peroxide-induced RCD (Schauer et al., 2009).

Mechanistically, acetic acid was shown to trigger vacuole membrane permeabilization and release of the vacuolar lumen protease Pep4p, the yeast CatD, while preserving vacuolar membrane integrity (Pereira et al., 2010). Deletion of *PEP4* in the W303-1B background was also reported to increase sensitivity to acetic acid, accompanied by an increase in clustered mitochondria and a delay in mitochondrial degradation. Overexpression had the opposite effect, suggesting that Pep4p and degradation of dysfunctional mitochondria have a protective role during acetic acid challenge (Pereira et al., 2010), later shown to depend on its proteolytic activity (Pereira et al., 2013). A similar protecting role was attributed to CatD in acetate-induced RCD in CRC cells (Oliveira et al., 2015). Although Pep4p is traditionally associated with bulk protein degradation in the vacuole (Takeshige et al., 1992), acetic acid did not induce autophagy either in yeast or in CRC cells (Pereira et al., 2010; Antonacci et al., 2012; Oliveira et al., 2015). Moreover, abrogation of autophagy via *ATG5* deletion had no effect on AA-RCD (Pereira et al., 2010). These results therefore indicate that the role of Pep4p in mitochondrial degradation and AA-RCD is independent of autophagy. Indeed, although a role in AA-RCD was attributed to the vacuolar membrane protein Atg22p, which belongs to the ATG family but is not necessary for autophagy, this was not attributed to any role in autophagy (Hu et al., 2019). The role of Pep4p was further linked with mitochondria by showing that increased sensitivity of *pep4*Δ cells to acetic acid requires the presence of Aac1/2/3 proteins, although not of Por1p (Pereira et al., 2013). Of note, in experiments using the BY4741 background, deletion of *PEP4* resulted in increased resistance, which could be due to the lower mitochondrial activity in this strain background (Sousa et al., 2013). Taken together, these data indicate that there is an intricate connection between the vacuole and mitochondria in AA-RCD involving the release of Pep4p, which mirrors the crosstalk between the lysosome and mitochondria in acetate-induced RCD in CRC cells and the release of CatD (Pereira et al., 2010; Antonacci et al., 2012; Marques et al., 2013; Oliveira et al., 2015).

### Lipid Regulators

Lipid metabolism plays a critical role in determining the membrane physical properties and in regulating the function of membrane-associated proteins, as well as in plasma membrane remodeling (Ziolkowska et al., 2012). Although these processes seem to be crucial to the acetic acid stress response, there are few studies on this topic in AA-RCD. It was shown that deletion of *GUP1*, a membrane-bound *O*-acyltransferase involved in a wide range of cellular processes such as lipid metabolism, glycosylphosphatidylinositol anchor remodeling, as well as in lipid rafts integrity and assembly, greatly reduces survival of yeast cells upon acetic acid challenge, pointing to the involvement of the membrane domains of lipid rafts in protection against AA-RCD (Tulha et al., 2012). Moreover, cells lacking Ysp2p, a mitochondria-localized sterol-binding protein that is responsible for the intracellular transport of sterols, were shown to be resistant to acetic acid (Sokolov et al., 2006). The involvement of sphingolipids in the regulation of AA-RCD has also been addressed, as alterations in the relative amounts of sphingolipids significantly affect cell fate, through activation of downstream effectors, modulation of protein trafficking, and activation of a variety of signaling pathways (Cowart and Obeid, 2007; Rego et al., 2014b). It has been suggested that ceramide production contributes to AA-RCD, especially through hydrolysis of complex sphingolipids catalyzed by Isc1p, the yeast inositol phosphosphingolipid phospholipase C and *de novo* synthesis catalyzed by Lag1p, the ceramide synthase (Rego et al., 2012). Absence of these two enzymes involved in ceramide production led to increased resistance to AA-RCD associated with lower levels of some phytoceramide species (described in more detail below) and reduced mitochondrial dysfunction, namely ROS accumulation, mitochondrial fragmentation and degradation, and cytochrome *c* release to the cytosol. In contrast, abrogation of the ceramide synthase Lac1p and the alkaline ceramidases Ydc1p and Ypc1p did not affect the survival of yeast cells (Rego et al., 2012). The interplay between sphingolipids, cell wall integrity signaling pathways and membrane remodeling in AA-RCD has also been explored and is discussed below. Lipidomic studies that further reinforce the role of sphingolipids in AA-RCD are also discussed.

### Genes Spanning Other Categories

Kex1p, a serine carboxypeptidase B-like protease, was suggested to, at least partially, mediate caspase-like activity in response to defects in N-glycosylation. A role for Kex1p in acetic acid-induced proteolytic activity was not ascertained, but deletion of *KEX1* resulted in decreased acetic acid-induced ROS accumulation; however, it resulted in only a slight increase in viability that was not statistically significant (Hauptmann and Lehle, 2008). One article assessed the role of peroxisomes in AA-RCD and showed that deletion of *PEX6*, encoding a protein involved in a key step of peroxisomal protein import, resulted in increased sensitivity to acetic acid. This was accompanied by increased ROS accumulation and PI staining. Authors thus concluded that deletion of *PEX6* causes necrotic cell death upon treatment with acetic acid, although a mechanism was not ascertained (Jungwirth et al., 2008).

## Signaling Pathways

**FIGURE 2:**
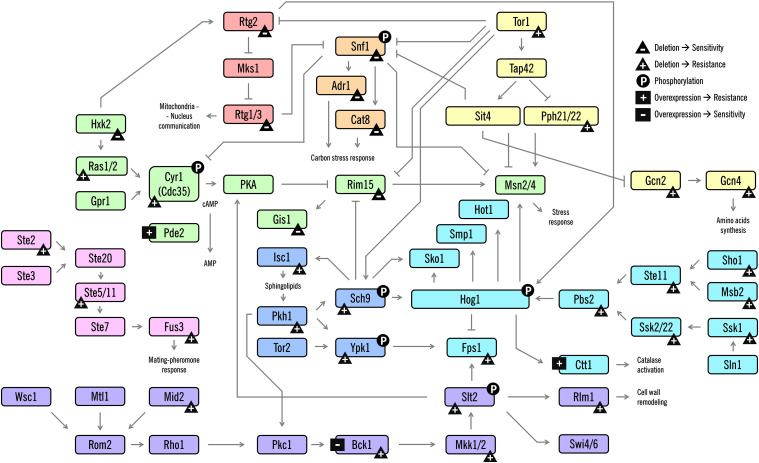
Schematic diagram of the signaling pathways involved in AA-RCD in yeast. Arrows indicate activation of proteins or promotion of a post-translational modification; lines with bars indicate their inhibition/inactivation. Different MAPK signaling pathways or clusters are color-coded as shown. The phenotypes resulting from the deletion or overexpression of the corresponding protein-coding gene are represented with symbols. The symbols are: triangle with a plus (gene deletion confers resistance), triangle with a minus (gene deletion confers sensitivity), square with a plus (protein overexpression confers resistance), and square with a minus (protein overexpression confers sensitivity). Phosphorylation is represented with a circle with a P. Some phenotypes were not included due to contradictory data among the studies. For clarity, despite the number of different proteins and signaling cascades displayed in this scheme, not all players, connections between the pathways and direct targets of the proteins are shown.

Cells coordinate a multifaceted response at many levels of physiology. They respond to stress by orchestrating complex responses critical for surviving stressful conditions. In yeast, these responses are typically regulated by upstream signaling pathways, most commonly mitogen-activated protein kinase (MAPK) cascades; these coexist in a cell to transduce signals, resulting in patterns of altered gene expression and protein activity to respond to the external environment (Saito, 2010). In the last decades, several studies have been conducted to unveil regulatory networks activated by acetic acid, as well as how the signal integration of the physiological processes is coordinated. In this section, we focus on the progress towards understanding these signaling responses during AA-RCD in yeast, focusing on the role of several signaling kinases, feedback mechanisms and important players of crosstalk between the pathways (Figure 2).

### PKH1-SCH9-YPK1 Signaling

Several signaling proteins have been shown to mediate the effects of sphingolipids and/or to regulate sphingolipid levels, mitochondria function and oxidative stress in response to several stimuli. These include the serine/threonine kinase Pkh1p, ortholog of the mammalian 3-phosphoinositide-dependent kinase PDK1 and an important regulator of the yeast sphingolipid biosynthetic pathway, which controls sphingolipid balance through Sch9p [yeast ortholog of mammalian protein kinase B (Akt/PKB)] and Ypk1p [yeast ortholog of mammalian serum- and glucocorticoid-inducible protein kinase (SGK)]. Acetic acid exposure was shown to trigger Pkh1/2p-dependent phosphorylation of both Sch9p and Ypk1p, demonstrating that Pkh1p-Ypk1p and Pkh1p-Sch9p pathways are activated during AA-RCD (Rego et al., 2020). The contribution of these signaling pathways and how sphingolipid signaling regulates AA-RCD, mainly with Isc1p as a downstream target, was then addressed in more detail. On one hand, single and double mutants lacking Isc1p or/and Sch9p had the same resistant phenotype, and *SCH9* deletion impaired acetic acid-induced translocation of Isc1p from the endoplasmic reticulum to mitochondria (Rego et al., 2018). These results suggest that the Sch9p pathway modulates AA-RCD through the regulation of Isc1p cellular distribution, thus affecting the sphingolipid balance that regulates cell fate. On the other hand, the Pkh1p-Ypk1p pathway seems necessary for the resistance to acetic acid displayed by *isc1*Δ cells (Rego et al., 2020). Indeed, in contrast with individual mutations, which caused resistance, absence of both *ISC1* and *PKH1* drastically reduced survival of yeast cells to acetic acid, accompanied by increased ROS accumulation and release of cytochrome *c* (Rego et al., 2020). However, this was likely associated with hyperactivation of the cAMP/PKA pathway and not due to a more specific role of the proteins in sphingolipid signaling (see next section).

### RAS-cAMP-PKA Signaling

In yeast, RAS acts upstream of acidification-induced cAMP/PKA signaling (Lastauskienë et al., 2014), and several studies indicate that the RAS/cAMP/PKA pathway is an important intracellular regulator of AA-RCD in yeast. *C. albicans* Ras1p and adenylate cyclase Cdc35p, which is involved in Ras-mediated signaling, were found to modulate cell death triggered by acetic acid, since *ras1*Δ *and cdc35*Δ cells are resistant to acetic acid (Phillips et al., 2006). Accordingly, overactivation of the pathway by the Ras1^Val13^ mutation or addition of dibutyryl-cAMP or cAMP-stimulatory drugs (caffeine targeting phosphodiesterase, forskolin activating adenylate cyclase) accelerated AA-CD as assessed by TUNEL assay, while addition of lovastatin (which blocks Ras farnesylation and its membrane localization), decreased it. *S. cerevisiae* cells lacking Ras2p, the main regulator of the cAMP/PKA pathway in this yeast, showed increased survival after the exposure to high doses (300–400 mM) of acetic acid in comparison with wild-type cells (Burtner et al., 2009), suggesting that Ras2p promotes AA-CD. In agreement, Amigoni et al. demonstrated that acetic acid causes re-localization of active Ras from the plasma membrane and nucleus to the mitochondria, which authors suggested contributes to AA-RCD (Amigoni et al., 2013). Consistent with this hypothesis, cells lacking the hexokinase Hxk2p, which display constitutive mitochondrial localization of active Ras (Broggi et al., 2013), showed increased AA-RCD as evidenced by a decrease in clonogenic survival, increased ROS accumulation, mitochondrial transmembrane potential, mitochondrial fragmentation and PS exposure in an Yca1p-independent manner (Amigoni et al., 2013). Expression of an active Ras mutant (Ras^Val19^), also increased acetic acid-induced ROS accumulation in *hxk2*Δ cells (Amigoni et al., 2013). In addition, the increased AA-RCD of *hxk2*Δ cells was reverted by deletion of *AIF1*, which abrogated the localization of active Ras at mitochondria (Amigoni et al., 2016). Of note, another report showed that deletion of *HXK2* did not enhance AA-CD when compared to the wild-type strain, though Ras localization was not assessed (Laera et al., 2016). Apart from a possible role in mitochondria, Ras activation may promote AA-RCD by increasing cAMP levels. Overexpression of *PDE2*, which encodes a high-affinity cAMP phosphodiesterase that inhibits PKA by hydrolyzing cAMP, increased the resistance of yeast cells to acetic acid (Rego et al., 2020). On the other hand, excessive levels of cAMP seem to result in sensitivity to acetic acid. Indeed, *isc1*Δ *pkh1*Δ double mutants display features associated with increased cAMP levels and their sensitivity to acetic acid was completely reverted by *PDE2* overexpression (Rego et al., 2020). Accordingly, abrogation of Snf1p, which when active phosphorylates and inhibits adenylate cyclase, thus resulting in lower cAMP levels and lower PKA activity (Nicastro et al., 2015), significantly reduced survival of yeast cells exposed to acetic acid (Bonomelli et al., 2020). This was accompanied by Ras accumulation in mitochondria (correlated with mitochondrial dysfunction) and a higher level of ROS, demonstrating an anti-apoptotic role for Snf1p that may be associated with decreased cAMP levels. Downstream of these proteins, absence of the glucose-repressible protein kinase Rim15p increased sensitivity to acetic acid (Burtner et al., 2009). This phenotype may however be due to several signaling pathways since, apart from PKA, Rim15p also integrates signals from nutrient-sensory kinases TORC1 and Sch9p (Swinnen et al., 2006). Indeed, the resistance of *sch9*Δ cells to acetic acid mentioned above was shown to depend on Rim15p and its downstream effector Gis1p (Burtner et al., 2009).

### HOG1-RTG2 Signaling

Exposure of yeast cells to acetic acid was first reported to activate the High-Osmolarity Glycerol (HOG) pathway and lead to increased tolerance to acetic acid on rich medium plates. However, exposure to 100 mM of acetate at pH 4.5 had no effect on *GPD1* mRNA, which encodes glycerol-3-phosphate dehydrogenase, and glycerol production, hallmarks of HOG pathway activation by hyperosmotic stress (Mollapour and Piper, 2006). Subsequently, it was reported that Hog1p directly phosphorylates the aquaglyceroporin channel Fps1p, targeting it for ubiquitination, endocytosis, and final degradation in the vacuole. Deletion of *FPS1* abolished the accumulation of undissociated acetic acid within the cells, and the authors correlated the loss of Fps1p with enhanced tolerance to acetic acid on solid media, though, again, death-inducing conditions were not tested (Mollapour and Piper, 2007; Mollapour et al., 2009). In a later report, deletion of *HOG1* did not affect the survival of yeast cells exposed to apoptosis-inducing concentrations of acetic acid, but several mutants of the HOG signaling pathway (*sho1*Δ, *msb2*Δ, *ssk1*Δ, *ssk22*Δ, and *pbs*2Δ) were significantly more resistant to AA-RCD than the wild type strain (Rego et al., 2014a). Since the HOG pathway is controlled through a strong feedback regulation, it seems that there is not always an obvious relationship between Hog1p response and the phenotype, and that the extent of the stress influences Hog1p activation and consequently the cellular outcome. Indeed, Guaragnella et al. reported that deletion of *HOG1* increased sensitivity of acid-adapted cells to AA-RCD, accompanied by increased DNA fragmentation and ROS accumulation. They further documented that adaptation to acid stress leads to Hog1p phosphorylation, which is delayed in the *rtg2*Δ mutant, and that both Hog1p and Rtg2p contribute to protection from AA-RCD imparted by acid adaptation, with the former being essential (Guaragnella et al., 2019). Furthermore, up-regulation of *CTT1* mRNA triggered by acid stress was diminished in *rtg2*Δ cells and drastically reduced in *hog1*Δ cells. This led authors to suggest that Rtg2p functions upstream of Hog1p to activate *CTT1* expression, imparting resistance to acetic acid through protection from oxidative stress and preservation of mitochondrial function. Because acid stress did not increase the expression of the Rtg1/3p target *CIT2*, they concluded this function was independent of retrograde response activation mediated by Rtg1/Rtg3 transcription (Guaragnella et al., 2019).

### TOR Signaling

The first evidence indicating TOR (target of rapamycin) signaling plays a role in AA-RCD came from a study comparing the total cellular proteome of acetic acid-treated and non-treated early stationary cells (Almeida et al., 2009). That study revealed that acetic acid induces severe intracellular amino acid starvation, involving the TOR pathway through the phosphatases Pph21p and Pph22p, but not Sit4p. Indeed, these authors observed a large increase in the survival of *tor1*Δ cells that was associated with a decrease in the percentage of TUNEL-positive and ROS-positive cells. *pph21*Δ and *pph22*Δ mutants were also highly resistant, while *sit4*Δ cells were slightly resistant to acetic acid. Deletion of *TOR1* also prevented acetic acid-induced decrease of several proteins, such as the ribonucleotide reductase components Rnr2p and Rnr4p, and the translation initiation proteins Tif1p/Tif2p. They further reported that deletion of *GCN2* also prevented the decrease of translation initiation (Tif1p/Tif2p) and elongation factors (Eft1p/Eft2p, Tef1p/Tef2p) in response to acetic acid and that, like *tor1*Δ, both *gcn4*Δ and *gcn2*Δ mutants were highly resistant to acetic acid, confirming an involvement of the TOR/GNC pathway in AA-RCD. Later on, Büttner et al. also associated the resistance phenotype of *tor1*Δ with a decrease in ROS accumulation (Büttner et al., 2011). On the other hand, another study suggests that Sit4p has a protective role in AA-CD, since deletion of *SIT4* decreased cell survival of wild-type yeast cells, though in this case early exponential cells were used (Rego et al., 2020), illustrating the complexity of the regulation network. Together, these studies suggest that signaling pathways that regulate carbohydrate and nitrogen metabolism both contribute to the modulation of AA-RCD.

### Cell Wall Integrity Signaling

Cells sense cell wall stress via a family of cell-surface sensors (Wsc1p and Mid1/2p). These activate a kinase cascade (Rom2p-Rho1p-Pkc1p), which in turn integrates the signals into Slt2p via the MAP kinase module composed of Bck1p and Mkk1/2p proteins. Subsequently, phosphorylated Slt2p enters the nucleus and activates the transcription factors Rlm1p and Swi4p/Swi6p, leading to the induction of genes involved in the Cell Wall Integrity (CWI) pathway responsible for maintenance and function of the yeast cell wall. It was reported that overactivation of the CWI pathway through overexpression of *BCK1* sensitizes cells to AA-RCD, and that several mutants in CWI components were significantly more resistant to acetic acid than the wild-type strain, such as *mid2*Δ, *bck1*Δ, *mkk1*Δ, *mkk2*Δ, *slt2*Δ, and *rlm1*Δ (Rego et al., 2014a). Additionally, *bck1*Δ, *slt2*Δ and *rlm1*Δ strains displayed decreased acetic acid-induced ROS accumulation and cytochrome *c* release, confirming the involvement of the CWI pathway in AA-RCD. Although in that study phosphorylation of Slt2p in response to acetic acid was not observed (Rego et al., 2014a), previous studies had shown Slt2p phosphorylation in response to lower concentrations of acetic acid (likely sub-lethal) (Mollapour and Piper, 2006). Rego et al. further screened strains mutated in all the non-essential genes under Rlm1p control regarding their phenotype in response to acetic acid, and found that deletion of some Rlm1p target genes resulted in resistance (those involved in cell wall remodeling), whereas deletion of others resulted in sensitivity (those involved in cell wall stability). Since the *rlm1*Δ mutant is resistant to AA-RCD, it appears that the main Rlm1p-mediated response to acetic acid is cell wall remodeling and thus that decreased cell wall remodeling is particularly important for acetic acid resistance (Rego et al., 2014a). This topic was recently further explored in a study aiming to understand the role of Atg22p in AA-RCD, where it was described that deletion and overexpression of *ATG22* delays and enhances AA-RCD, respectively (Hu et al., 2019). Of note, authors showed that *atg22*Δ mutants exhibit higher cell wall integrity than the wild-type strain after acetic acid treatment by counteracting the reduction of glucan, mannan and chitin, and upregulating the expression of key genes of the CWI pathway. They also demonstrated that *ATG22* deletion has a protective effect at the level of membrane integrity, fluidity and permeability upon acetic acid exposure, displaying a change in the composition of phospholipids, sterols and fatty acids, suggesting that the effect of Atg22p in CWI may be pleiotropic (Hu et al., 2019).

### Other Signaling Pathways

Deficiency in several components of the mating-pheromone response pathway resulted in higher resistance to AA-RCD. This included the *ste2*Δ, *ste11*Δ and *ste5*Δ and *fus3*Δ mutants, which exhibited lower accumulation of ROS and a higher number of cells with plasma membrane integrity than wild-type cells upon acetic acid exposure. On the other hand, the invasive growth/pseudohyphal development signaling pathway does not seem to be involved in AA-RCD, since deletion of *KSS1* had no effect on yeast cells exposed to acetic acid (Rego et al., 2014a).

## High Throughput Studies

A wider and comprehensive view of the mechanisms occurring during AA-RCD has been obtained using high throughput assays, both at genomic, transcriptomic, proteomic and metabolomic levels.

Genome-wide identification of genes involved in the positive and negative regulation of AA-RCD in *S. cerevisiae* was performed using a functional analysis of an Euroscarf yeast knock-out haploid mutant collection. This study identified known pro-apoptotic and anti-apoptotic genes and confirmed mitochondrial function as the most relevant category in modulation of cell death induced by acetic acid (Sousa et al., 2013). It also identified transcription of glucose-repressed genes, protein synthesis and modifications, as well as vesicular traffic, as important for protection against AA-RCD. On the other hand, amino acid transport and biosynthesis, oxidative stress response, cell growth and differentiation, protein phosphorylation and histone deacetylation stood out as important for the execution of AA-RCD. The second most significantly enriched term in the resistant strain dataset was “Metabolic process.” This dataset comprised strains deficient in genes involved in amino acid metabolism (the most represented), central carbohydrate metabolism (including glycolysis, fermentation, citric acid cycle and pentose-phosphate pathway) and metabolism of C2 compounds, suggesting that the slowdown of energy production pathways can be beneficial to decrease cell death, in agreement with the dependence of an active cell death process on energy. These results also evidenced the important impact of metabolic status on AA-RCD, both at the level of carbohydrate, lipid or amino acid metabolism, and established a connection between cell proliferation and cell death regulation (Sousa et al., 2013). The regulated nature of this cell death process, both at the level of protein phosphorylation and epigenetic regulation by acetylation, was further evidenced among the most represented terms in the positive regulation of AA-RCD. These included a high number of genes coding for protein phosphatases and kinases (mainly from MAPK signaling pathways and in the regulation of metabolism, cell cycle, budding, cell polarity and filament formation), as well as all the genes coding for the components of the Set3 histone deacetylase complex (Sousa et al., 2013), in agreement with earlier and subsequent studies discussed in the previous sections.

Several works assessed transcriptional changes after exposure to acetic acid, however under different conditions. Most used established sub-lethal concentrations of acetic acid or cells after adaptation and are reviewed elsewhere (Lee et al., 2015; Palma et al., 2018). One study assessed cell death in parallel, ensuring the chosen concentration and times corresponded to death-inducing conditions (Dong et al., 2017). This approach also allowed a comparison between the different time points. Overall, genes with altered transcription were involved in biological pathways similar to those found in the above-mentioned chemogenomics screen: “cellular homeostasis, central metabolic pathway, stress response, transcription regulation and histone modification, cellular uptake and transport, ubiquitination process, protein synthesis, MAPK signaling pathway, cell cycle and DNA repair, and programmed cell death.” Authors found that four dominant biological processes emerged: heat shock protein, amino acid metabolism, ribosome and carbohydrate metabolism. Indeed, these categories seem to be the most represented in most of the omics studies, even those assessing responses to adaptation or sub-lethal acetic acid concentrations (Kawahata et al., 2006; Abbott et al., 2007; Li and Yuan, 2010; Mira et al., 2010; Bajwa et al., 2013; Sousa et al., 2013; Lee et al., 2015), and additional metabolomic studies are in agreement with these results. For instance, gas chromatography-mass spectrometry showed that acetic acid has varying impacts on amino acid carbohydrate and lipid metabolism; there was a clear decrease in amino acids, as well as down-regulation of metabolites related to carbohydrate metabolism (Dong et al., 2017). Through analysis of two-dimensional gel electrophoresis (2-DE) spots, Almeida et al. had already found that acetic acid affects the levels of stress response proteins, mostly chaperones, as well as proteins involved in the transcription/translation machinery, amino acid and nucleotide biosynthesis and carbohydrate metabolism, with most proteins showing reduced levels. These authors also reported that acetic acid induces severe intracellular amino acid starvation (Almeida et al., 2009). In contrast, Longo et al. showed an increase in the levels of multiple amino acids in wild-type cells but amino acid depletion in *yca1*Δ cells. Cell death-inducing conditions were used by Longo et al. (2015), and thus it is not clear what prompted the opposing results, especially since changes in protein levels assessed by 2-DE were similar to those found by Almeida et al. (2009). Proteomic analysis was also performed in mitochondrial-enriched fractions of *Z. parabailii* ISA1307 under conditions that lead to AA-RCD, with maintenance of plasma membrane integrity, DNA fragmentation, cytochrome *c* release and ROS accumulation (Guerreiro et al., 2016). In this study, the most representative functional classes reported as important for AA-RCD were also energy and carbohydrate metabolism, amino acid metabolism and protein folding, in agreement with results from *S. cerevisiae*. Of note, some proteins involved in translation were also decreased, such as Tef2p and Ola1p, but other proteins involved in ribosome biogenesis increased in acetic acid-treated cells. It has however been suggested that general translation is inhibited but translation of specific mRNAs is selectively increased. Indeed, microarray analysis of mRNAs translated during acetic acid treatment of *S. cerevisiae* cells showed a decrease in overall polysome-associated mRNAs, though a few mRNAs showed increased association with polysomes at 15 and 30 min (Silva et al., 2013). These mainly belonged to the categories “cellular protein metabolic process, cytoplasm and organelle organization, translation regulation and ribosome structure,” suggesting that acetic acid exposure leads to remodeling of the cellular content and translation process. Authors further analyzed two of these, the HSP90 isoforms *HSC82* and *HSP82*, and found that increased expression of Hsc82p and Hsp82p led to higher resistance and sensitivity to acetic acid, respectively. In agreement, deletion of Hsc82p resulted in higher sensitivity to acetic acid, accompanied by an increase in PI-staining but not in TUNEL-positive cells, whereas deletion of Hsp82p resulted in higher resistance. These results suggest that HSP90 chaperones play a dual role in the modulation of AA-RCD, which authors speculate could be due to distinct protein interactions (Silva et al., 2013). Following up on these studies, Samanfar et al. screened an array of 384 strains deficient in proteins involved in protein synthesis to identify those that could affect sensitivity to acetic acid by influencing translation control in a mRNA 5’ CAP-independent manner. They found that deletion of *DOM34* or *RPL36A* increased sensitivity to acetic acid, and decreased translation of Hsp82p, though not affecting mRNA levels. They further showed that overexpression of *RPL36A* or *DOM34* rescued acetic acid sensitivity of several strains lacking genes involved in ribosome biogenesis (listing mutants in a number of different genes, but only showing results for *rpl43a*Δ and *rps29b*Δ), and conclude that *DOM34* and *RPL36A* influence HSP82-5’-UTR mediated translation in response to acetic acid (Samanfar et al., 2017).

Finally, lipidomic analysis of sphingolipid species by high-performance liquid chromatography/mass spectrometry (LC-MS/MS) suggested that ceramide production contributes to AA-RCD. Indeed, wild-type yeast cells exposed to acetic acid showed increased levels of phytosphingosine (PHS), phytoceramides Phyto-C18-Cer and α-OH-Phyto-C20-Cer and decreased levels of phosphorylated long chain bases (LCBPs), dihydrosphingosine-1-phosphate (DHS-1-P) and phytosphingosine-1-phosphate (PHS-1-P) (Rego et al., 2012). Similarly, a decrease in the levels of DHS-1-P, PHS and PHS-1-P and an increase of most phytoceramide and dihydroceramide species was observed in *yca1*Δ cells upon acetic acid exposure (Longo et al., 2015). In contrast, *isc1*Δ and *lag1*Δ mutants, which are resistant to acetic acid, showed lower levels of some phytoceramide species (Phyto-C16-Cer and Phyto-C18-Cer and α-OH-Phyto-C20-Cer) and increased levels of levels of DHS-1-P and PHS-1-P (Rego et al., 2012). When mitochondrial sphingolipid species were analyzed, it was also found that a higher resistance of yeast cells to acetic acid correlates with higher levels of endogenous mitochondrial LCBPs. In that study, analysis of the different dihydroceramides and phytoceramide species did not reveal any in particular that increased in the wild-type but not in the resistant mutant cells tested (*isc1*Δ, *sch9*Δ and *isc1*Δ *sch9*Δ). On the other hand, levels of both DHS-1-P and/or PHS-1-P, associated with a pro-survival role, decreased in mitochondria of wild-type cells but increased in the mutants (Rego et al., 2018). Overall, those results suggest that changing the sphingolipid balance in favor of LCBPs in mitochondria and whole cells results in increased survival to acetic acid.

## Discussion and Concluding Remarks

Acetic acid has been extensively used as a lethal stimulus in yeast RCD studies, which allowed characterization of the role of different molecular components and organelles involved in cell death commitment in response to this acid. Mechanisms by which the death process can be regulated have also been uncovered, and further knowledge on this subject may therefore impact different biotechnological applications. At first glance, understanding the mechanisms underlying AA-RCD may seem to lack industrial relevance, since sub-lethal concentrations of acetic acid can already inhibit industrial bioprocesses and lethal acetic acid concentrations would render the process inviable. However, on one hand, the observed growth in the presence of acetic acid concentrations often present in industrial fermentation media is already the net result of growth and death of yeast cells. On the other hand, yeast cells do face high concentrations of acetic acid in industrial applications, in particular in biorefineries employing lignocellulosic substrates. Indeed, acetic acid is often present in these substrates at concentrations that do not allow growth and are lethal for wild type strains. As such, the acquired knowledge on AA-RCD will allow manipulating this process to construct robust strains with improved fermentation performance.

Many candidate targets for improving the resistance of industrial strains have been identified so far, opening an array of opportunities for the construction of better performing strains. Attractive targets are the regulation of catabolic repression genes and modification of mitochondrial or vacuolar activity, which, as aforementioned, have already begun to be explored. Nonetheless, care must be taken so that the alterations introduced do not affect the fermentative performance of the strains, in order not to compromise their potential for industrial application. This is particularly relevant as “decreased replication” is an ontology term significantly associated with resistance to AA-RCD (Sousa et al., 2013).

The multifactorial nature of AA-RCD may complicate the development of industrially robust strains, emphasizing the need to further pursue research on this topic. However, a few practical examples of the exploitation of mechanisms involved in AA-RCD either by manipulating its players or associated processes, which resulted in a successful strain improvement, have already been reported. For instance, Kumar et al. overexpressed *COX20*, coding for a mitochondrial cytochrome *c* oxidase chaperone, obtaining improved tolerance to acetic acid during fermentation (Kumar et al., 2015). In fact, increased respiration had been associated with resistance to AA-RCD both in early cell death studies (Ludovico et al., 2002; Pereira et al., 2007) and by omics approaches (Sousa et al., 2013). In another study by Balderas-Hernández et al., removal of the general catabolite repressor Mig1p resulted in increased yeast growth and ethanol production in anaerobic batch fermentations in the presence of inhibitory concentrations of acetic acid (Balderas-Hernández et al., 2018), which is consistent with the reported increased sensitivity to AA-RCD under glucose repression conditions reported by multiple authors. Deletion of *RTT109*, coding for a histone acetyltransferase responsible for the acetylation of histone H3, was also shown to shorten the lag phase and increase growth and ethanol production rates in fermentation medium with 5 g/L (≈ 80 mM) acetic acid (Cheng et al., 2016). This result shows that epigenetic regulation, with a prominent role of in AA-RCD, also impacts fermentation performance. The cell wall integrity pathway is another process that has been clearly identified as a determinant of AA-RCD, with the impairment of cell wall remodeling associated with increased resistance (Sousa et al., 2013; Rego et al., 2014a). Accordingly, increased cell wall resistance has been successfully targeted by overexpressing *PRS3* and *HAA1* in a xylose-utilizing *S. cerevisiae* strain, and resulted in improved consumption of both glucose and xylose (Cunha et al., 2018).

On another note, the use of acetic acid as an inducer of regulated cell death in yeast has also identified several molecular players that can be further investigated in mammalian systems, where the apoptotic machinery is yet to be fully characterized. For instance, studies have shown that acetate is able to preferentially activate the intrinsic pathway of apoptosis in CRC cell lines over normal cell types (Jan et al., 2002), and further research has already determined the role of some components of the mammalian apoptotic process based on yeast-based studies. One example is the mediation of mitochondrial degradation by the yeast Pep4p (Pereira et al., 2007) which is mimicked by its mammalian ortholog CatD (Oliveira et al., 2015). As such, unraveling the molecular interplay that underlies acetic acid-induced cell death is of great biomedical interest: not only for the possible use of acetate as a prophylactic or therapeutic agent in tumorigenesis, but also, in a broader sense, to better characterize and aid in a plethora of diseases where apoptosis is dysregulated.

In summary, the twenty-year long research on AA-RCD in yeast has allowed the identification and characterization of the role of different cellular components, organelles and signaling pathways involved in this process, as well as the mechanisms through which it can be regulated. The knowledge here presented not only reinforces the power of this unicellular eukaryotic organism as a versatile and robust model system toward improved biomedical solutions, but also exposes multiple candidate targets for improving industrial strains. As these targets have only begun to be explored, this field is wide open for future exploitation.

## Author Contributions

All authors wrote sections of the manuscript, contributed to the article, and approved the submitted version.

## Conflict of Interest

The authors declare that the research was conducted in the absence of any commercial or financial relationships that could be construed as a potential conflict of interest.

## References

[B1] Abbott D. A. Knijnenburg T. A. De Poorter L. M. Reinders M. J. Pronk J. T. Van Maris A. J. (2007). Generic and specific transcriptional responses to different weak organic acids in anaerobic chemostat cultures of *Saccharomyces cerevisiae*. *FEMS Yeast Res.* 7 819–833. 10.1111/j.1567-1364.2007.00242.x 17484738

[B2] Aerts A. M. Carmona-Gutierrez D. Lefevre S. Govaert G. François I. E. Madeo F. (2009). The antifungal plant defensin RsAFP2 from radish induces apoptosis in a metacaspase independent way in Candida albicans. *FEBS Lett.* 583 2513–2516. 10.1016/j.febslet.2009.07.004 19596007

[B3] Agus H. H. Kok G. Derinoz E. Oncel D. Yilmaz S. (2020). Involvement of Pca1 in ROS-mediated apoptotic cell death induced by alpha-thujone in the fission yeast (*Schizosaccharomyces pombe*). *FEMS Yeast Res.* 20:foaa022. 10.1093/femsyr/foaa022 32347926

[B4] Almeida B. Ohlmeier S. Almeida A. J. Madeo F. Leão C. Rodrigues F. (2009). Yeast protein expression profile during acetic acid-induced apoptosis indicates causal involvement of the TOR pathway. *Proteomics* 9 720–732. 10.1002/pmic.200700816 19137548

[B5] Alugoju P. Periyasamy L. Dyavaiah M. (2018). Quercetin enhances stress resistance in *Saccharomyces cerevisiae tel1* mutant cells to different stressors. *J. Food Sci. Technol.* 55 1455–1466. 10.1007/s13197-018-3062-9 29606760 PMC5876216

[B6] Amigoni L. Frigerio G. Martegani E. Colombo S. (2016). Involvement of Aif1 in apoptosis triggered by lack of Hxk2 in the yeast *Saccharomyces cerevisiae*. *FEMS Yeast Res.* 16:fow016. 10.1093/femsyr/fow016 26895787

[B7] Amigoni L. Martegani E. Colombo S. (2013). Lack of HXK2 induces localization of active Ras in mitochondria and triggers apoptosis in the yeast *Saccharomyces cerevisiae*. *Oxid Med. Cell. Longev.* 2013:678473. 10.1155/2013/678473 24089630 PMC3780702

[B8] Antonacci L. Guaragnella N. Ždralevic M. Passarella S. Marra E. Giannattasio S. (2012). The N-acetylcysteine-insensitive acetic acid-induced yeast programmed cell death occurs without macroautophagy. *Curr. Pharm. Biotechnol.* 13 2705–2711. 10.2174/138920112804724819 23072389

[B9] Bajwa P. K. Ho C. Y. Chan C. K. Martin V. J. Trevors J. T. Lee H. (2013). Transcriptional profiling of *Saccharomyces cerevisiae* T2 cells upon exposure to hardwood spent sulphite liquor: comparison to acetic acid, furfural and hydroxymethylfurfural. *Antonie Van Leeuwenhoek* 103 1281–1295. 10.1007/s10482-013-9909-1 23539198

[B10] Balderas-Hernández V. E. Correia K. Mahadevan R. (2018). Inactivation of the transcription factor mig1 (YGL035C) in *Saccharomyces cerevisiae* improves tolerance towards monocarboxylic weak acids: acetic, formic and levulinic acid. *J. Ind. Microbiol. Biotechnol.* 45 735–751. 10.1007/s10295-018-2053-1 29876685

[B11] Bhat T. A. Chaudhary A. K. Kumar S. O’malley J. Inigo J. R. Kumar R. (2017). Endoplasmic reticulum-mediated unfolded protein response and mitochondrial apoptosis in cancer. *Biochim. Biophys. Acta Rev. Cancer* 1867 58–66. 10.1016/j.bbcan.2016.12.002 27988298 PMC5272864

[B12] Bonomelli B. Martegani E. Colombo S. (2020). Lack of SNF1 induces localization of active Ras in mitochondria and triggers apoptosis in the yeast *Saccharomyces cerevisiae*. *Biochem. Biophys. Res. Commun.* 523 130–134. 10.1016/j.bbrc.2019.12.023 31837801

[B13] Broggi S. Martegani E. Colombo S. (2013). Live-cell imaging of endogenous Ras-GTP shows predominant Ras activation at the plasma membrane and in the nucleus in *Saccharomyces cerevisiae*. *Int. J. Biochem. Cell. Biol.* 45 384–394. 10.1016/j.biocel.2012.10.013 23127800

[B14] Burtner C. R. Murakami C. J. Kennedy B. K. Kaeberlein M. (2009). A molecular mechanism of chronological aging in yeast. *Cell Cycle* 8 1256–1270. 10.4161/cc.8.8.8287 19305133 PMC2746416

[B15] Büttner S. Eisenberg T. Carmona-Gutierrez D. Ruli D. Knauer H. Ruckenstuhl C. (2007). Endonuclease G regulates budding yeast life and death. *Mol. Cell.* 25 233–246. 10.1016/j.molcel.2006.12.021 17244531

[B16] Büttner S. Ruli D. Vögtle F. N. Galluzzi L. Moitzi B. Eisenberg T. (2011). A yeast BH3-only protein mediates the mitochondrial pathway of apoptosis. *EMBO J.* 30 2779–2792. 10.1038/emboj.2011.197 21673659 PMC3160254

[B17] Camougrand N. Grelaud-Coq A. Marza E. Priault M. Bessoule J. J. Manon S. (2003). The product of the UTH1 gene, required for Bax-induced cell death in yeast, is involved in the response to rapamycin. *Mol. Microbiol.* 47 495–506. 10.1046/j.1365-2958.2003.03311.x 12519199

[B18] Cardoso H. Leão C. (1992). Mechanisms underlying the low and high enthalphy death induced by short-chain monocarboxylic acids and ethanol in *Saccharomyces cerevisiae*. *Appl. Microb. Cell Physiol.* 38 388–392. 10.1007/BF00170091

[B19] Carmelo V. Santos H. Sá-Correia I. (1997). Effect of extracellular acidification on the activity of plasma membrane ATPase and on the cytosolic and vacuolar pH of *Saccharomyces cerevisiae*. *Biochim. Biophys. Acta* 1325 63–70. 10.1016/s0005-2736(96)00245-3 9106483

[B20] Carmona-Gutierrez D. Bauer M. A. Zimmermann A. Aguilera A. Austriaco N. Ayscough K. (2018). Guidelines and recommendations on yeast cell death nomenclature. *Microb. Cell* 5 4–31. 10.15698/mic2018.01.607 29354647 PMC5772036

[B21] Casal M. Cardoso H. Leao C. (1996). Mechanisms regulating the transport of acetic acid in *Saccharomyces cerevisiae*. *Microbiology* 142 1385–1390. 10.1099/13500872-142-6-1385 8704978

[B22] Cássio F. Leão C. Van Uden N. (1987). Transport of lactate and other short-chain monocarboxylates in the yeast *Saccharomyces cerevisiae*. *Appl. Environ. Microbiol.* 53 509–513. 10.1128/aem.53.3.509-513.1987 3034152 PMC203697

[B23] Cebulski J. Malouin J. Pinches N. Cascio V. Austriaco N. (2011). Yeast bax inhibitor, Bxi1p, is an ER-localized protein that links the unfolded protein response and programmed cell death in *Saccharomyces cerevisiae*. *PLoS One* 6:e20882. 10.1371/journal.pone.0020882 21673967 PMC3108976

[B24] Cheng C. Zhao X. Zhang M. Bai F. (2016). Absence of Rtt109p, a fungal-specific histone acetyltransferase, results in improved acetic acid tolerance of *Saccharomyces cerevisiae*. *FEMS Yeast Res.* 16:fow010. 10.1093/femsyr/fow010 26851403

[B25] Cheng W. C. Teng X. Park H. K. Tucker C. M. Dunham M. J. Hardwick J. M. (2008). Fis1 deficiency selects for compensatory mutations responsible for cell death and growth control defects. *Cell Death Differ.* 15 1838–1846. 10.1038/cdd.2008.117 18756280 PMC2631236

[B26] Conrad M. Schothorst J. Kankipati H. N. Van Zeebroeck G. Rubio-Texeira M. Thevelein J. M. (2014). Nutrient sensing and signaling in the yeast *Saccharomyces cerevisiae*. *FEMS Microbiol. Rev.* 38 254–299. 10.1111/1574-6976.12065 24483210 PMC4238866

[B27] Cosentino K. García-Sáez A. J. (2014). Mitochondrial alterations in apoptosis. *Chem. Phys. Lipids* 181 62–75. 10.1016/j.chemphyslip.2014.04.001 24732580

[B28] Cowart L. A. Obeid L. M. (2007). Yeast sphingolipids: recent developments in understanding biosynthesis, regulation, and function. *Biochim. Biophys. Acta* 1771 421–431. 10.1016/j.bbalip.2006.08.005 16997623 PMC1868558

[B29] Cunha J. T. Costa C. E. Ferraz L. Romaní A. Johansson B. Sá-Correia I. (2018). HAA1 and PRS3 overexpression boosts yeast tolerance towards acetic acid improving xylose or glucose consumption: unravelling the underlying mechanisms. *Appl. Microbiol. Biotechnol.* 102 4589–4600. 10.1007/s00253-018-8955-z 29607452

[B30] Deprez M. A. Eskes E. Wilms T. Ludovico P. Winderickx J. (2018). pH homeostasis links the nutrient sensing PKA/TORC1/Sch9 ménage-à-trois to stress tolerance and longevity. *Microb. Cell* 5 119–136. 10.15698/mic2018.03.618 29487859 PMC5826700

[B31] Di Domenico M. Martínez A. Lana P. Worsaae K. (2014). Molecular and morphological phylogeny of *Saccocirridae* (Annelida) reveals two cosmopolitan clades with specific habitat preferences. *Mol. Phylogenet. Evol.* 75 202–218. 10.1016/j.ympev.2014.02.003 24561268

[B32] Dilova I. Chen C. Y. Powers T. (2002). Mks1 in concert with TOR signaling negatively regulates RTG target gene expression in S. cerevisiae. *Curr. Biol.* 12 389–395. 10.1016/s0960-9822(02)00677-2 11882290

[B33] Dong Y. Hu J. Fan L. Chen Q. (2017). RNA-Seq-based transcriptomic and metabolomic analysis reveal stress responses and programmed cell death induced by acetic acid in *Saccharomyces cerevisiae*. *Sci. Rep.* 7:42659. 10.1038/srep42659 28209995 PMC5314350

[B34] Fannjiang Y. Cheng W. C. Lee S. J. Qi B. Pevsner J. Mccaffery J. M. (2004). Mitochondrial fission proteins regulate programmed cell death in yeast. *Genes Dev.* 18 2785–2797. 10.1101/gad.1247904 15520274 PMC528898

[B35] Giannattasio S. Atlante A. Antonacci L. Guaragnella N. Lattanzio P. Passarella S. (2008). Cytochrome c is released from coupled mitochondria of yeast en route to acetic acid-induced programmed cell death and can work as an electron donor and a ROS scavenger. *FEBS Lett.* 582 1519–1525. 10.1016/j.febslet.2008.03.048 18396162

[B36] Giannattasio S. Guaragnella N. Corte-Real M. Passarella S. Marra E. (2005). Acid stress adaptation protects *Saccharomyces cerevisiae* from acetic acid-induced programmed cell death. *Gene* 354 93–98. 10.1016/j.gene.2005.03.030 15894436

[B37] Guaragnella N. Antonacci L. Giannattasio S. Marra E. Passarella S. (2008). Catalase T and Cu,Zn-superoxide dismutase in the acetic acid-induced programmed cell death in *Saccharomyces cerevisiae*. *FEBS Lett.* 582 210–214. 10.1016/j.febslet.2007.12.007 18082141

[B38] Guaragnella N. Antonacci L. Passarella S. Marra E. Giannattasio S. (2007). Hydrogen peroxide and superoxide anion production during acetic acid-induced yeast programmed cell death. *Folia Microbiol. (Praha)* 52 237–240. 10.1007/bf02931304 17702461

[B39] Guaragnella N. Bobba A. Passarella S. Marra E. Giannattasio S. (2010a). Yeast acetic acid-induced programmed cell death can occur without cytochrome c release which requires metacaspase YCA1. *FEBS Lett.* 584 224–228. 10.1016/j.febslet.2009.11.072 19941863

[B40] Guaragnella N. Passarella S. Marra E. Giannattasio S. (2010b). Knock-out of metacaspase and/or cytochrome c results in the activation of a ROS-independent acetic acid-induced programmed cell death pathway in yeast. *FEBS Lett.* 584 3655–3660. 10.1016/j.febslet.2010.07.044 20674572

[B41] Guaragnella N. Passarella S. Marra E. Giannattasio S. (2011). Cytochrome c Trp65Ser substitution results in inhibition of acetic acid-induced programmed cell death in *Saccharomyces cerevisiae*. *Mitochondrion* 11 987–991. 10.1016/j.mito.2011.08.007 21907312

[B42] Guaragnella N. Pereira C. Sousa M. J. Antonacci L. Passarella S. Corte-Real M. (2006). YCA1 participates in the acetic acid induced yeast programmed cell death also in a manner unrelated to its caspase-like activity. *FEBS Lett.* 580 6880–6884. 10.1016/j.febslet.2006.11.050 17156780

[B43] Guaragnella N. Stirpe M. Marzulli D. Mazzoni C. Giannattasio S. (2019). Acid stress triggers resistance to acetic acid-induced regulated cell death through. *Oxid. Med. Cell. Longev* 2019:4651062. 10.1155/2019/4651062 30931079 PMC6410445

[B44] Guaragnella N. Ždralević M. Lattanzio P. Marzulli D. Pracheil T. Liu Z. (2013). Yeast growth in raffinose results in resistance to acetic-acid induced programmed cell death mostly due to the activation of the mitochondrial retrograde pathway. *Biochim. Biophys. Acta* 1833 2765–2774.1. 10.1016/j.bbamcr.2013.07.017 23906793

[B45] Guerreiro J. F. Sampaio-Marques B. Soares R. Coelho A. V. Leão C. Ludovico P. (2016). Mitochondrial proteomics of the acetic acid - induced programmed cell death response in a highly tolerant. *Microb. Cell* 3 65–78.1. 10.15698/mic2016.02.477 28357336 PMC5349105

[B46] Hague A. Elder D. J. Hicks D. J. Paraskeva C. (1995). Apoptosis in colorectal tumour cells: induction by the short chain fatty acids butyrate, propionate and acetate and by the bile salt deoxycholate. *Int. J. Cancer* 60 400–406.1. 10.1002/ijc.2910600322 7829251

[B47] Hauptmann P. Lehle L. (2008). Kex1 protease is involved in yeast cell death induced by defective N-glycosylation, acetic acid, and chronological aging. *J. Biol. Chem.* 283 19151–19163.1. 10.1074/jbc.M801303200 18474590

[B48] Heerdt B. G. Houston M. A. Augenlicht L. H. (1997). Short-chain fatty acid-initiated cell cycle arrest and apoptosis of colonic epithelial cells is linked to mitochondrial function. *Cell Growth Differ.* 8 523–532. 9149903

[B49] Hill S. M. Nyström T. (2015). The dual role of a yeast metacaspase: what doesn’t kill you makes you stronger. *Bioessays* 37 525–531.1. 10.1002/bies.201400208 25677381 PMC5053244

[B50] Hu J. Dong Y. Wang W. Zhang W. Lou H. Chen Q. (2019). Deletion of *Atg22* gene contributes to reduce programmed cell death induced by acetic acid stress in *Saccharomyces cerevisiae*. *Biotechnol. Biofuels* 12:298. 10.1186/s13068-019-1638-x 31890026 PMC6933646

[B51] Hüttemann M. Pecina P. Rainbolt M. Sanderson T. H. Kagan V. E. Samavati L. (2011). The multiple functions of cytochrome c and their regulation in life and death decisions of the mammalian cell: From respiration to apoptosis. *Mitochondrion* 11 369–381.1. 10.1016/j.mito.2011.01.010 21296189 PMC3075374

[B52] Jan G. Belzacq A. S. Haouzi D. Rouault A. Métivier D. Kroemer G. (2002). Propionibacteria induce apoptosis of colorectal carcinoma cells via short-chain fatty acids acting on mitochondria. *Cell Death Differ.* 9 179–188.1. 10.1038/sj.cdd.4400935 11840168

[B53] Jungwirth H. Ring J. Mayer T. Schauer A. Büttner S. Eisenberg T. (2008). Loss of peroxisome function triggers necrosis. *FEBS Lett.* 582 2882–2886. 10.1016/j.febslet.2008.07.023 18656474

[B54] Kalyanaraman B. Darley-Usmar V. Davies K. J. Dennery P. A. Forman H. J. Grisham M. B. (2012). Measuring reactive oxygen and nitrogen species with fluorescent probes: challenges and limitations. *Free Radic. Biol. Med.* 52 1–6. 10.1016/j.freeradbiomed.2011.09.030 22027063 PMC3911769

[B55] Kawahata M. Masaki K. Fujii T. Iefuji H. (2006). Yeast genes involved in response to lactic acid and acetic acid: acidic conditions caused by the organic acids in *Saccharomyces cerevisiae* cultures induce expression of intracellular metal metabolism genes regulated by Aft1p. *FEMS Yeast Res.* 6 924–936. 10.1111/j.1567-1364.2006.00089.x 16911514

[B56] Kawazoe N. Kimata Y. Izawa S. (2017). Acetic acid causes endoplasmic reticulum stress and induces the unfolded protein response in *Saccharomyces cerevisiae*. *Front. Microbiol.* 8:1192. 10.3389/fmicb.2017.01192 28702017 PMC5487434

[B57] Komeili A. Wedaman K. P. O’shea E. K. Powers T. (2000). Mechanism of metabolic control. Target of rapamycin signaling links nitrogen quality to the activity of the Rtg1 and Rtg3 transcription factors. *J. Cell Biol.* 151 863–878. 10.1083/jcb.151.4.863 11076970 PMC2169436

[B58] Kumar V. Hart A. J. Keerthiraju E. R. Waldron P. R. Tucker G. A. Greetham D. (2015). Expression of mitochondrial cytochrome C oxidase chaperone gene (COX20) improves tolerance to weak acid and oxidative stress during yeast fermentation. *PLoS One* 10:e0139129. 10.1371/journal.pone.0139129 26427054 PMC4591339

[B59] Laera L. Guaragnella N. Ždralević M. Marzulli D. Liu Z. Giannattasio S. (2016). The transcription factors *ADR1* or *CAT8* are required for RTG pathway activation and evasion from yeast acetic acid-induced programmed cell death in raffinose. *Microb. Cell* 3 621–631. 10.15698/mic2016.12.549 28357334 PMC5348981

[B60] Lastauskienë E. Zinkevićienë A. Girkontaitë I. Kaunietis A. Kvedarienë V. (2014). Formic acid and acetic acid induce a programmed cell death in pathogenic Candida species. *Curr. Microbiol.* 69 303–310. 10.1007/s00284-014-0585-9 24752490

[B61] Leão C. Van Uden N. (1986). Transport of lactate and other short-chain monocarboxylates in the yeast *Candida utilis*. *Appl. Microbiol. Biotechnol.* 23 339–389. 10.1007/BF00257039 PMC203697 3034152

[B62] Lee R. E. Brunette S. Puente L. G. Megeney L. A. (2010). Metacaspase Yca1 is required for clearance of insoluble protein aggregates. *Proc. Natl. Acad. Sci. U S A* 107 13348–13353.1. 10.1073/pnas.1006610107 20624963 PMC2922170

[B63] Lee Y. Nasution O. Choi E. Choi I. G. Kim W. Choi W. (2015). Transcriptome analysis of acetic-acid-treated yeast cells identifies a large set of genes whose overexpression or deletion enhances acetic acid tolerance. *Appl. Microbiol. Biotechnol.* 99 6391–6403. 10.1007/s00253-015-6706-y 26062532

[B64] Légiot A. Céré C. Dupoiron T. Kaabouni M. Camougrand N. Manon S. (2019). Mitochondria-Associated Membranes (MAMs) are involved in Bax mitochondrial localization and cytochrome c release. *Microb. Cell* 6 257–266. 10.15698/mic2019.05.678 31114795 PMC6506693

[B65] Li B. Z. Yuan Y. J. (2010). Transcriptome shifts in response to furfural and acetic acid in *Saccharomyces cerevisiae*. *Appl. Microbiol. Biotechnol.* 86 1915–1924. 10.1007/s00253-010-2518-2 20309542

[B66] Liu C. Apodaca J. Davis L. E. Rao H. (2007). Proteasome inhibition in wild-type yeast *Saccharomyces cerevisiae* cells. *Biotechniques* 42:158. 10.2144/000112389 17373478

[B67] Longo V. Zdralevic M. Guaragnella N. Giannattasio S. Zolla L. Timperio A. M. (2015). Proteome and metabolome profiling of wild-type and YCA1-knock-out yeast cells during acetic acid-induced programmed cell death. *J. Proteomics* 128 173–188. 10.1016/j.jprot.2015.08.003 26269384

[B68] Ludovico P. Rodrigues F. Almeida A. Silva M. T. Barrientos A. Corte-Real M. (2002). Cytochrome *c* release and mitochondria involvement in programmed cell death induced by acetic acid in *Saccharomyces cerevisiae*. *Mol. Biol. Cell* 13 2598–2606. 10.1091/mbc.e01-12-0161 12181332 PMC117928

[B69] Ludovico P. Sansonetty F. Silva M. T. Côrte-Real M. (2003). Acetic acid induces a programmed cell death process in the food spoilage yeast Zygosaccharomyces bailii. *FEMS Yeast Res.* 3 91–96. 10.1111/j.1567-1364.2003.tb00143.x 12702251

[B70] Ludovico P. Sousa M. J. Silva M. T. Leão C. L. Côrte-Real M. (2001). *Saccharomyces cerevisiae* commits to a programmed cell death process in response to acetic acid. *Microbiology* 147 2409–2415. 10.1099/00221287-147-9-2409 11535781

[B71] Ma F. Zhang Y. Wang Y. Wan Y. Miao Y. Ma T. (2016). Role of Aif1 in regulation of cell death under environmental stress in Candida albicans. *Yeast* 33 493–506. 10.1002/yea.3167 27121326

[B72] Madeo F. Eisenberg T. Pietrocola F. Kroemer G. (2018). Spermidine in health and disease. *Science* 359:eaan2788. 10.1126/science.aan2788 29371440

[B73] Madeo F. Herker E. Maldener C. Wissing S. Lächelt S. Herlan M. (2002). A caspase-related protease regulates apoptosis in yeast. *Mol. Cell* 9 911–917. 10.1016/s1097-2765(02)00501-4 11983181

[B74] Marchetti C. Migliorati G. Moraca R. Riccardi C. Nicoletti I. Fabiani R. (1997). Deoxycholic acid and SCFA-induced apoptosis in the human tumor cell-line HT-29 and possible mechanisms. *Cancer Lett.* 114 97–99. 10.1016/s0304-3835(97)04634-x 9103263

[B75] Marques C. Oliveira C. S. Alves S. Chaves S. R. Coutinho O. P. Côrte-Real M. (2013). Acetate-induced apoptosis in colorectal carcinoma cells involves lysosomal membrane permeabilization and cathepsin D release. *Cell Death Dis.* 4:e507. 10.1038/cddis.2013.29 23429293 PMC3734821

[B76] Martins V. M. Fernandes T. R. Lopes D. Afonso C. B. Domingues M. R. M. Côrte-Real M. (2019). Contacts in death: the role of the ER-Mitochondria axis in acetic acid-induced apoptosis in yeast. *J. Mol. Biol.* 431 273–288. 10.1016/j.jmb.2018.11.002 30414966

[B77] Matouschek A. Rospert S. Schmid K. Glick B. S. Schatz G. (1995). Cyclophilin catalyzes protein folding in yeast mitochondria. *Proc. Natl. Acad. Sci. U S A* 92 6319–6323. 10.1073/pnas.92.14.6319 7603990 PMC41509

[B78] Mazzoni C. Falcone C. (2008). Caspase-dependent apoptosis in yeast. *Biochim. Biophys. Acta* 1783 1320–1327. 10.1016/j.bbamcr.2008.02.015 18355456

[B79] Michel A. H. Kornmann B. (2012). The ERMES complex and ER-mitochondria connections. *Biochem. Soc. Trans.* 40 445–450. 10.1042/bst20110758 22435828

[B80] Mira N. P. Becker J. D. Sá-Correia I. (2010). Genomic expression program involving the Haa1p-regulon in *Saccharomyces cerevisiae* response to acetic acid. *OMICS* 14 587–601. 10.1089/omi.2010.0048 20955010 PMC3125556

[B81] Mollapour M. Piper P. W. (2006). Hog1p mitogen-activated protein kinase determines acetic acid resistance in *Saccharomyces cerevisiae*. *FEMS Yeast Res.* 6 1274–1280. 10.1111/j.1567-1364.2006.00118.x 17156024

[B82] Mollapour M. Piper P. W. (2007). Hog1 mitogen-activated protein kinase phosphorylation targets the yeast Fps1 aquaglyceroporin for endocytosis, thereby rendering cells resistant to acetic acid. *Mol. Cell Biol.* 27 6446–6456. 10.1128/mcb.02205-06 17620418 PMC2099610

[B83] Mollapour M. Shepherd A. Piper P. W. (2009). Presence of the Fps1p aquaglyceroporin channel is essential for Hog1p activation, but suppresses Slt2(Mpk1)p activation, with acetic acid stress of yeast. *Microbiology* 155 3304–3311. 10.1099/mic.0.030502-0 19608606

[B84] Mroczek S. Kufel J. (2008). Apoptotic signals induce specific degradation of ribosomal RNA in yeast. *Nucleic Acids Res.* 36 2874–2888. 10.1093/nar/gkm1100 18385160 PMC2396418

[B85] Nicastro R. Tripodi F. Gaggini M. Castoldi A. Reghellin V. Nonnis S. (2015). Snf1 phosphorylates adenylate cyclase and negatively regulates protein kinase A-dependent transcription in *Saccharomyces cerevisiae*. *J. Biol. Chem.* 290 24715–24726. 10.1074/jbc.m115.658005 26309257 PMC4598984

[B86] Oliveira C. S. Pereira H. Alves S. Castro L. Baltazar F. Chaves S. R. (2015). Cathepsin D protects colorectal cancer cells from acetate-induced apoptosis through autophagy-independent degradation of damaged mitochondria. *Cell Death Dis.* 6:e1788. 10.1038/cddis.2015.157 26086961 PMC4669836

[B87] Palma M. Guerreiro J. F. Sá-Correia I. (2018). Adaptive response and tolerance to acetic acid in *Saccharomyces cerevisiae* and *Zygosaccharomyces bailii*: a physiological genomics perspective. *Front. Microbiol.* 9:274. 10.3389/fmicb.2018.00274 29515554 PMC5826360

[B88] Parikh V. S. Morgan M. M. Scott R. Clements L. S. Butow R. A. (1987). The mitochondrial genotype can influence nuclear gene expression in yeast. *Science* 235 576–580. 10.1126/science.3027892 3027892

[B89] Pereira C. Camougrand N. Manon S. Sousa M. J. Côrte-Real M. (2007). ADP/ATP carrier is required for mitochondrial outer membrane permeabilization and cytochrome c release in yeast apoptosis. *Mol. Microbiol.* 66 571–582. 10.1111/j.1365-2958.2007.05926.x 17822411

[B90] Pereira C. Chaves S. Alves S. Salin B. Camougrand N. Manon S. (2010). Mitochondrial degradation in acetic acid-induced yeast apoptosis: the role of Pep4 and the ADP/ATP carrier. *Mol. Microbiol.* 76 1398–1410. 10.1111/j.1365-2958.2010.07122.x 20345665

[B91] Pereira H. Azevedo F. Rego A. Sousa M. J. Chaves S. R. Côrte-Real M. (2013). The protective role of yeast cathepsin D in acetic acid-induced apoptosis depends on ANT (Aac2p) but not on the voltage-dependent channel (Por1p). *FEBS Lett.* 587 200–205. 10.1016/j.febslet.2012.11.025 23220089

[B92] Phillips A. J. Crowe J. D. Ramsdale M. (2006). Ras pathway signaling accelerates programmed cell death in the pathogenic fungus Candida albicans. *Proc. Natl. Acad. Sci. U S A* 103 726–731. 10.1073/pnas.0506405103 16407097 PMC1334641

[B93] Pinto I. Cardoso H. Leão C. Van Uden N. (1989). High enthalpy and low enthalpy death in *Saccharomyces cerevisiae* induced by Acetic Acid. *Biotechnol. Bioeng.* 33 1350–1352. 10.1002/bit.260331019 18587871

[B94] Prudêncio C. Sansonetty F. Côrte-Real M. (1998). Flow cytometric assessment of cell structural and functional changes induced by acetic acid in the yeasts *Zygosaccharomyces bailii* and *Saccharomyces cerevisiae*. *Cytometry* 31 307–313. 10.1002/(sici)1097-0320(19980401)31:4<307::aid-cyto11<3.0.co;2-u 9551607

[B95] Rego A. Cooper K. F. Snider J. Hannun Y. A. Costa V. Côrte-Real M. (2018). Acetic acid induces Sch9p-dependent translocation of Isc1p from the endoplasmic reticulum into mitochondria. *Biochim. Biophys. Acta Mol. Cell. Biol. Lipids* 1863 576–583. 10.1016/j.bbalip.2018.02.008 29496584 PMC5899942

[B96] Rego A. Costa M. Chaves S. R. Matmati N. Pereira H. Sousa M. J. (2012). Modulation of mitochondrial outer membrane permeabilization and apoptosis by ceramide metabolism. *PLoS One* 7:e48571. 10.1371/journal.pone.0048571 23226203 PMC3511487

[B97] Rego A. Duarte A. M. Azevedo F. Sousa M. J. Côrte-Real M. Chaves S. R. (2014a). Cell wall dynamics modulate acetic acid-induced apoptotic cell death of. *Microb. Cell.* 1 303–314. 10.15698/mic2014.09.164 28357256 PMC5349133

[B98] Rego A. Trindade D. Chaves S. R. Manon S. Costa V. Sousa M. J. (2014b). The yeast model system as a tool towards the understanding of apoptosis regulation by sphingolipids. *FEMS Yeast Res.* 14 160–178. 10.1111/1567-1364.12096 24103214

[B99] Rego A. Mendes F. Costa V. Chaves S. R. Côrte-Real M. (2020). Pkh1p-Ypk1p and Pkh1p-Sch9p pathways are activated by acetic acid to induce a mitochondrial-dependent regulated cell death. *Oxid. Med. Cell Longev.* 2020:7095078. 10.1155/2020/7095078 32318242 PMC7154982

[B100] Ribeiro G. F. Côrte-Real M. Johansson B. (2006). Characterization of DNA damage in yeast apoptosis induced by hydrogen peroxide, acetic acid, and hyperosmotic shock. *Mol. Biol. Cell* 17 4584–4591. 10.1091/mbc.e06-05-0475 16899507 PMC1635349

[B101] Ribéreau-Gayon P. Dubourdieu D. Donèche B. Lonvaud A. (2006). *”Alcohols and other volatile compounds,” in Handbook of Enology: The Microbiology of Wine and Vinifications*, 2nd Edn. Hoboken, NJ: John Wiley & Sons, 51–64.

[B102] Saito H. (2010). Regulation of cross-talk in yeast MAPK signaling pathways. *Curr. Opin. Microbiol.* 13 677–683. 10.1016/j.mib.2010.09.001 20880736

[B103] Samanfar B. Shostak K. Moteshareie H. Hajikarimlou M. Shaikho S. Omidi K. (2017). The sensitivity of the yeast, *Saccharomyces cerevisiae*, to acetic acid is influenced by *DOM34* and *RPL36A*. *PeerJ.* 5:e4037. 10.7717/peerj.4037 29158977 PMC5691786

[B104] Saraiva L. Silva R. D. Pereira G. Gonçalves J. Côrte-Real M. (2006). Specific modulation of apoptosis and Bcl-xL phosphorylation in yeast by distinct mammalian protein kinase C isoforms. *J. Cell Sci.* 119 3171–3181. 10.1242/jcs.03033 16835272

[B105] Schauer A. Knauer H. Ruckenstuhl C. Fussi H. Durchschlag M. Potocnik U. (2009). Vacuolar functions determine the mode of cell death. *Biochim. Biophys. Acta* 1793 540–545. 10.1016/j.bbamcr.2008.11.006 19100296

[B106] Scheppach W. Bartram H. P. Richter F. (1995). Role of short-chain fatty acids in the prevention of colorectal cancer. *Eur. J. Cancer* 31A 1077–1080. 10.1016/0959-8049(95)00165-f 7576995

[B107] Sekito T. Liu Z. Thornton J. Butow R. A. (2002). RTG-dependent mitochondria-to-nucleus signaling is regulated by MKS1 and is linked to formation of yeast prion [URE3]. *Mol. Biol. Cell* 13 795–804.1. 10.1091/mbc.01-09-0473 11907262 PMC99599

[B108] Semchyshyn H. M. Abrat O. B. Miedzobrodzki J. Inoue Y. Lushchak V. I. (2011). Acetate but not propionate induces oxidative stress in bakers’ yeast *Saccharomyces cerevisiae*. *Redox Rep.* 16 15–23. 10.1179/174329211x12968219310954 21605494 PMC6837459

[B109] Silva A. Sampaio-Marques B. Fernandes A. Carreto L. Rodrigues F. Holcik M. (2013). Involvement of yeast HSP90 isoforms in response to stress and cell death induced by acetic acid. *PLoS One* 8:e71294. 10.1371/journal.pone.0071294 23967187 PMC3744546

[B110] Simões-Mendes B. Madeira-Lopes A. Van Uden N. (1978). Kinetics of petite mutation and thermal death in *Saccharomyces cerevisiae* growing at superoptimal temperatures. *Z Allg. Mikrobiol.* 18 275–279. 10.1002/jobm.19780180406 354226

[B111] Sj S. Veerabhadrappa B. Subramaniyan S. Dyavaiah M. (2019). Astaxanthin enhances the longevity of *Saccharomyces cerevisiae* by decreasing oxidative stress and apoptosis. *FEMS Yeast Res.* 19:foy113. 10.1093/femsyr/foy113 30312390

[B112] Sokolov S. Knorre D. Smirnova E. Markova O. Pozniakovsky A. Skulachev V. (2006). Ysp2 mediates death of yeast induced by amiodarone or intracellular acidification. *Biochim. Biophys. Acta* 1757 1366–1370. 10.1016/j.bbabio.2006.07.005 16962064

[B113] Sousa M. Duarte A. M. Fernandes T. R. Chaves S. R. Pacheco A. Leão C. (2013). Genome-wide identification of genes involved in the positive and negative regulation of acetic acid-induced programmed cell death in *Saccharomyces cerevisiae*. *BMC Genom.* 14:838. 10.1186/1471-2164-14-838 24286259 PMC4046756

[B114] Sousa M. J. Ludovico P. Rodrigues F. Leão C. Côrte-Real M. (2012). *”Stress and cell death in yeast induced by acetic acid,” in Cell Metabolism – Cell Homeostasis and Stress Response.* Princes Gate Court, UK: InTech. 10.5772/27726

[B115] Sousa M. J. Miranda L. Côrte-Real M. Leão C. (1996). Transport of acetic acid in *Zygosaccharomyces bailii*: effects of ethanol and their implications on the resistance of the yeast to acidic environments. *Appl. Environ. Microbiol.* 62 3152–3157.1. 10.1128/AEM.62.9.3152-3157.1996 8795203 PMC168109

[B116] Suen D. F. Norris K. L. Youle R. J. (2008). Mitochondrial dynamics and apoptosis. *Genes Dev.* 22 1577–1590.1. 10.1101/gad.1658508 18559474 PMC2732420

[B117] Susin S. A. Lorenzo H. K. Zamzami N. Marzo I. Snow B. E. Brothers G. M. (1999). Molecular characterization of mitochondrial apoptosis-inducing factor. *Nature* 397 441–446.1. 10.1038/17135 9989411

[B118] Swinnen E. Wanke V. Roosen J. Smets B. Dubouloz F. Pedruzzi I. (2006). Rim15 and the crossroads of nutrient signalling pathways in *Saccharomyces cerevisiae*. *Cell. Div.* 1:3. 10.1186/1747-1028-1-3 16759348 PMC1479807

[B119] Taherzadeh M. J. Eklund R. Gustafsson L. Niklasson C. Lidén G. (1997). Characterization and fermentation of Dilute-Acid Hydrolyzates from wood. *Indus. Eng. Chem. Res.* 36 4659–4665. 10.1021/ie9700831

[B120] Takeshige K. Baba M. Tsuboi S. Noda T. Ohsumi Y. (1992). Autophagy in yeast demonstrated with proteinase-deficient mutants and conditions for its induction. *J. Cell. Biol.* 119 301–311.1. 10.1083/jcb.119.2.301 1400575 PMC2289660

[B121] Teng X. Dayhoff-Brannigan M. Cheng W. C. Gilbert C. E. Sing C. N. Diny N. L. (2013). Genome-wide consequences of deleting any single gene. *Mol. Cell.* 52 485–494.1. 10.1016/j.molcel.2013.09.026 24211263 PMC3975072

[B122] Toth A. Aufschnaiter A. Fedotovskaya O. Dawitz H. Ädelroth P. Büttner S. (2020). Membrane-tethering of cytochrome c accelerates regulated cell death in yeast. *Cell Death Dis.* 11:722. 10.1038/s41419-020-02920-0 32892209 PMC7474732

[B123] Trindade D. Pereira C. Chaves S. R. Manon S. Côrte-Real M. Sousa M. J. (2016). VDAC regulates AAC-mediated apoptosis and cytochrome. *Microb. Cell.* 3 500–510. 10.15698/mic2016.10.533 28357318 PMC5348984

[B124] Tulha J. Faria-Oliveira F. Lucas C. Ferreira C. (2012). Programmed cell death in *Saccharomyces cerevisiae* is hampered by the deletion of GUP1 gene. *BMC Microbiol.* 12:80. 10.1186/1471-2180-12-80 22617017 PMC3444424

[B125] Váchová L. Palková Z. (2007). Caspases in yeast apoptosis-like death: facts and artefacts. *FEMS Yeast Res.* 7 12–21.1. 10.1111/j.1567-1364.2006.00137.x 17311581

[B126] Valenti D. Vacca R. A. Guaragnella N. Passarella S. Marra E. Giannattasio S. (2008). A transient proteasome activation is needed for acetic acid-induced programmed cell death to occur in *Saccharomyces cerevisiae*. *FEMS Yeast Res.* 8 400–404.1. 10.1111/j.1567-1364.2008.00348.x 18218016

[B127] Vall-Llaura N. Mir N. Garrido L. Vived C. Cabiscol E. (2019). Redox control of yeast Sir2 activity is involved in acetic acid resistance and longevity. *Redox Biol.* 24:101229. 10.1016/j.redox.2019.101229 31153040 PMC6543126

[B128] Van Opdenbosch N. Lamkanfi M. (2019). Caspases in cell death, inflammation, and disease. *Immunity* 50 1352–1364.1. 10.1016/j.immuni.2019.05.020 31216460 PMC6611727

[B129] Van Uden N. (1984). Temperature profiles of yeasts. *Adv. Microb. Physiol.* 25 195–251.1. 10.1016/s0065-2911(08)60293-3 6398621

[B130] Wissing S. Ludovico P. Herker E. Buttner S. Engelhardt S. M. Decker T. (2004). An AIF orthologue regulates apoptosis in yeast. *J. Cell Biol.* 166 969–974.1. 10.1083/jcb.200404138 15381687 PMC2172025

[B131] Wu H. Ng B. S. Thibault G. (2014). Endoplasmic reticulum stress response in yeast and humans. *Biosci. Rep.* 34:e00118. 10.1042/BSR20140058 24909749 PMC4076835

[B132] Zamora F. (2009). “Biochemistry of alcoholic fermentation,” in *Wine Chemistry and Biochemistry*, eds Moreno-Arribas M. V. Polo M. C. (New York: Springer), 3–26. 10.1007/978-0-387-74118-5_1

[B133] Ždralević M. Longo V. Guaragnella N. Giannattasio S. Timperio A. M. Zolla L. (2015). Differential proteome-metabolome profiling of YCA1-knock-out and wild type cells reveals novel metabolic pathways and cellular processes dependent on the yeast metacaspase. *Mol. Biosyst.* 11 1573–1583.1. 10.1039/C4MB00660G 25697364

[B134] Ziolkowska N. E. Christiano R. Walther T. C. (2012). Organized living: formation mechanisms and functions of plasma membrane domains in yeast. *Trends Cell Biol.* 22 151–158. 10.1016/j.tcb.2011.12.002 22245053

