# Supplementary material for: Regulation of Cell Death Induced by Acetic Acid in Yeasts
Source: Front Cell Dev Biol. 2021 Jun 24;9:642375. doi: 10.3389/fcell.2021.642375 (PMC8264433; doi:10.3389/fcell.2021.642375)
Supplement: Supplementary file 1 [file Data_Sheet_1.PDF]

Supplementary Material

Supplementary Tables

Table S1: Bibliographic compilation of studies on the characterisation of AA-RCD in yeasts, cellular markers monitored and respective methodology. A summary of methods and results interpretations is listed as reported by the authors. Please refer to the individual manuscripts for details regarding protocols and controls. **2-DE**: Bidimensional Electrophoresis; **Ac-FCS**: Ac-IETD/VEID-AMC, fluorogenic caspase-8/caspase-6 substrate; **A-DY**: Angel High Active Dry Yeast; **AO/EtBr**: Acridine Orange/Ethidium Bromide; **AV**: Annexin V; **BD/TLC/PQ**: Bligh & Dyer Method/Thin-layer Chromatography/Phosphorus Quantification; **Ca**: *C. albicans*; **CLbGFP**: GFP carrying the presequence of mitochondrial citrate synthase; **Cg/Cl + Ca 10231**: *C. guilliermondii* & *C. lusitaniae* clinical isolates + *C. albicans* ATCC 10231; **COE**: Clark Oxygen Electrode; **Cox4-GFP**: matrix-targeted domain of cytochrome oxidase subunit IV (Cox4) fused to GFP; **DAPI:** 4′,6-diamidino-2-phenylindole; **DHR**: Dihydrorhodamine 123; **DiOC**: $DiOC_6(3)$, 3,3′dihexyloxacarbocyanine iodide; **DiSC**: $DiSC_3(3)$, 3,3′-Dipropylthiacarbocyanine iodide; **DHE**: Dihydroethidium; **FC**: Flow Cytometry; **FDA**: Fluorescein Diacetate; **F-VAD**: FITC-VAD-fmk, Fluorescein Isothiocyanate conjugate of the pan-caspase inhibitor VAD(OMe)-FMK; **FOX**: Xylenol Orange colorimetric assay; **FM**: Fluorescence Microscopy; **FR**: Fluorescence Reader; **FUN-1**: 2-chloro-4-(2,3-dihydro-3-methyl-(benzo-1,3-thiazol-2-yl)-methylidene)-1-phenylquinolinium iodide; **HFDA**: H_2DCFDA, 2',7'-dichlorodihydrofluorescein diacetate; **LCEC-MS/MS**: Liquid Chromatography Electrospray Ionization Collision-induced Dissociation Tandem Mass Spectrometry; **LC-MS/MS**: Liquid Chromatography Tandem Mass Spectrometry; **MTOF/TOF**: MALDI-TOF/TOF, Matrix-assisted laser desorption/ionization Time-Of-Flight Mass Spectrometry; **MTR**: MitoTracker Red CM-H_2XRos; **MS**: Mass Spectrometry; **mpHL**: (mt)pHluorin, (mitochondria-targeted) GFP that is sensitive to pH changes; **mtCFP**: matrix-targeted CFP; **mtGFP**: matrix-targeted GFP; **NB**: Northern Blot; **PFGE**: Pulse Field Gel Electrophoresis; **PI**: Propidium Iodide; **PM**: Phenotypical Microarray; **Rh**: Rhodamine 123; **RM**: RNA Microarray; **RTqPCR**: Reverse Transcription Quantitative Polymerase Chain Reaction; **RS**: Raman Spectra; **RXS**: Redox Spectrophotometry; **SEM**: Scanning Electron Microscopy; **SF**: Spectrofluorimeter; **SP**: Spectrophotometry; **Sp ED666**: *S. pombe* ED666; **SNARF**: SNARF-4F, 1,4(and 5)-benzenedicarboxylic acid, 2-[10-(dimethylamino)-4-fluoro-3-oxo-3H-benzo[c]xanthen-7-yl]; **TN**: Terminal Deoxynucleotidyl Transferase dUTP Nick End Labeling (TUNEL); **TEM**: Transmission Electron Microscopy; **WB**: Western Blot; **Zp**: *Zygosaccharomyces parabailii*; **zVAD**: z-VAD-fmk, pan-caspase inhibitor.

Strain	DNA fragmentation	Chromatin condensation	Phosphatidylserine exposure	Caspase activation	Reactive Oxygen Species accumulation	Organelle ultrastructural changes	Mitochondrial degradation/fragmentation	Cytochrome *c* release	Loss of plasma membrane integrity	Altered plasma or mitochondrial membrane potential	Cytosolic and/or mitochondrial matrix acidification	Reduced metabolic activity and/or inhibition of respiration	rRNA degradation	Translation impairment	Modulation of sphingolipid or phospholipid levels	Reference
Saccharomyces cerevisiae																
**W303-1A**	TN/PI FM	TEM	AV/PI FM			TEM										Ludovico et al., 2001
	TN/PI FM				MTR^{FC}			WB/ RXS		Rh$^{FC/FM}$		COE				Ludovico et al., 2002
													NB			Mroczek & Kufel, 2008
		DAPI FM	AV/PI FC		DHE^{FC}				PI^{FC}	DiOC FM						Tulha et al., 2012
			AV/PI FC		DHR^{FC}					DiOC FM/FC						Amigoni et al., 2013
					DHE^{FC}		mtGFP FC		PI^{FC}							Pereira et al., 2013
			AV/PI FC		DHR^{FC}											Amigoni et al., 2016
			AV/PI FC		DHR^{FC}											Bonomelli et al., 2020
**W303-1B**		DAPI FM		F-VAD/ PI^{FC}												Guaragnella et al., 2006
					HFDA/ DHE^{FM}											Guaragnella et al., 2007
	TN/PI FM	DAPI FM		F-VAD/ PI^{FC}	MTR^{FM}			WB/ RXS	PI^{FM}							Pereira et al., 2007
	TN/PI FM							WB				COE				Giannattasio et al., 2008
					HFDA FM											Guaragnella et al., 2008

**W303-1B**	TN/PI ^{FM}			Ac-FCS/ zVAD^{SF}	HFDA ^{FM}			WB								Guaragnella et al., 2010a
	TN/PI ^{FM}			Ac-FCS^{SF}	HFDA ^{FM}			WB								Guaragnella et al., 2010b
		DAPI ^{FM}					mtGFP $^{FM/FC}$ MTR^{FM} WB		PI^{FC}	DiOC ^{FC}						Pereira et al., 2010
	TN/PI ^{FM}			Ac-FCS^{SF}	HFDA ^{FM}			WB								Guaragnella et al., 2011
	TN/PI ^{FM}				HFDA ^{FM}			WB								Guaragnella et al., 2013
	TN/PI ^{FM}						mtGFP ^{FM}							2-DE/ LCEC- MS/MS		Longo et al., 2015
							mtGFP ^{FM}	WB/ RXS								Trindade et al., 2016
	TN/PI ^{FM}		AV/PI ^{FM}		HFDA ^{FM}											Laera et al., 2016
	TEM	TEM	AV/PI ^{FC}			SEM/ TEM	mtGFP^{FC}				mpHL					Dong et al., 2017
	TN/PI ^{FM}				HFDA ^{FM}											Guaragnella et al., 2019
**BY4741**			AV/PI ^{FM}			TEM	Cox4- GFP^{FM}					FUN-1 ^{FC}				Fannjiang et al., 2004
	TN/PI ^{FM} + PFGE															Ribeiro et al., 2006
					HFDA ^{FM}											Sokolov et al., 2006
			AV/PI ^{FC}		DHE^{FR}				PI^{FC}							Jungwirth et al., 2008
													NB			Mroczek & Kufel, 2008
	TN/PI ^{FC}		AV/PI ^{FC}		DHE$^{FC/FR}$				PI^{FC}		SNARF ^{FC}					Schauer et al., 2009
					DHE^{FC}			WB	PI^{FC}							Rego et al., 2014
												PM				Kumar et al., 2015
					DHR/ DHE^{FC}											Guerreiro et al., 2016
					FOX^{SP}				PI^{FM}							Lee et al., 2016
									PI^{FC}							Tulha & Lucas, 2018

**BY4741**									PI^{SF}	DiSC^{SF}						Godinho et al., 2018
		DAPI^{FM}							PI^{FM}							Alugoju et al., 2018a
		DAPI^{FM}	AO/EtBr^{FM}						PI^{FM}							Alugoju et al., 2018b
					DHE^{FC}		mtGFP^{FC}	WB	PI^{FC}	DiOC^{FC}					BD/TLC/PQ	Martins et al., 2019
	DAPI^{FM}	AO/EtBr^{FM}	AV/PI^{FM}													Sj et al., 2019
					DHE^{FC}			WB	PI^{FC}							Rego et al., 2020
**BY4742**	TN/PI^{FM}	DAPI^{FM}			DHR^{FM}									2-DE/MS/WB		Almeida et al., 2009
	TN/PI^{FM}								PI^{FM}					RTqPCR RM/WB		Silva et al., 2013
			AV/PI^{FC}		DHE^{FC}	TEM/SEM				Rh^{FC}					LC-MS	Hu et al., 2019
**IGC 4072**									PI^{FC}			FDA^{FC}				Prudêncio et al., 1998
**CG379**					MTR/DHE^{FC}		CLbGFP$^{FM/FC}$	WB	PI^{FC}						LC-MS/MS	Rego et al., 2012
					MTR/DHE^{FC}		mtCFP$^{FM/FC}$	WB							LC-MS/MS	Rego et al., 2018
**SS328**					DHR^{FM}											Hauptmann et al., 2008
**AHADY**		DAPI^{FM}						RS				RS				Li et al., 2015
**Other species**																
**Zp ISA 1307**									PI^{FC}			FDA^{FC}				Prudêncio et al., 1998
	TN/PI^{FM}	TEM							PI^{FC}	Rh$^{FC/FM}$						Ludovico et al., 2003
	TN/PI^{FM}				DHR/DHE^{FC}			WB				SP		2-DE/MTOF/TOF		Guerreiro et al., 2016

**Ca BWP17**				F-VAD^{FM}												Aerts et al., 2009
**Ca SC5314**	TN/PI ^{FM}								PI^{FM}							Phillips et al., 2006
**Cg/Cl + Ca 10231**	TN/PI ^{FM}		AV/PI ^{FM}	F-VAD^{FM}												Lastauskienė et al., 2014
**Sp ED666**		DAPI ^{FM}	AO/ EtBr^{FM}		HFDA^{FM}											Agus et al., 2020
