# Supplementary material for: Regulation of Cell Death Induced by Acetic Acid in Yeasts
Source: Front Cell Dev Biol. 2021 Jun 24;9:642375. doi: 10.3389/fcell.2021.642375 (PMC8264433; doi:10.3389/fcell.2021.642375)
Supplement: Supplementary file 2 [file Data_Sheet_2.pdf]

Supplementary Material

1 Supplementary Tables

Table S2. Genes involved in acetic acid-induced regulated cell death and respective cellular alterations studied. *Saccharomyces cerevisiae* strains (BY4741, BY4742, CG379, CML128, DBY746, SS328 and W303-1A/B) and non-*Saccharomyces* strains (*Candida albicans* strains SC5314 and BWP17, and *Schizosaccharomyces pombe* E6666) are depicted. PM: plasma membrane; PS: phosphatidylserine; ROS: reactive oxygen species; $\Delta\Psi_m$: mitochondrial transmembrane potential.

Gene	Gene function	Background strain	Genetic variation	Phenotype	Cellular alterations	Reference
AAC1/2/3	Mitochondrial ADP/ATP carrier isoforms	W303-1B	Deletion	Resistant	↓ Clonogenic survival<br>↓ Chromatin condensation<br>↓ Loss of PM integrity<br>↓ DNA fragmentation<br>= ROS accumulation<br>↓ Cytochrome *c* release	Pereira et al., 2007
					↓ Mitochondrial degradation<br>↓ $\Delta\Psi_m$	Pereira et al., 2010
					↑ Clonogenic survival<br>↓ Cytochrome *c* release	Trindade et al., 2016
ADA2	Histone modification	BY4742	Deletion	Sensitive	↑ PS exposure	Dong et al., 2017
		W303-1B	Overexpression	Sensitive	↑ PS exposure	Dong et al., 2017
ADR1	Carbon source-responsive zinc-finger transcription factor	W303-1B	Deletion	Sensitive	↓ Clonogenic survival<br>↑ PS exposure<br>↑ DNA fragmentation<br>↑ ROS accumulation	Laera et al., 2016
AHC2	Histone modification	BY4742	Deletion	Sensitive	↑ PS exposure	Dong et al., 2017
		W303-1B	Overexpression	Sensitive	↑ PS exposure	Dong et al., 2017
ATG22	Vacuolar integral membrane protein required for efflux of amino acids	BY4742	Deletion	Resistant	↓ Clonogenic survival<br>↓ PS exposure<br>↑ $\Delta\Psi_m$<br>↓ ROS accumulation	Hu et al., 2019
			Overexpression	Sensitive	↑ PS exposure<br>↑ Loss of PM integrity	Hu et al., 2019
ATP2	β subunit of the F1 part of F_OF_1-ATPase	W303-1B	Deletion	Sensitive	↓ Clonogenic survival	Pereira et al., 2007
ATP10	ATP synthase assembly factor	W303-1A	Deletion	Resistant	↑ Clonogenic survival<br>↓ Cytochrome *c* release	Ludovico et al., 2002

BCK1	MAPKKK acting in the PKC signaling pathway	BY4741	Deletion	Resistant	↑ Clonogenic survival<br>↓ Loss of PM integrity<br>↓ ROS accumulation<br>↓ Cytochrome *c* release	Rego et al., 2014
			Overexpression	Sensitive	↓ Clonogenic survival	Rego et al., 2014
CAT8	Zinc cluster transcriptional activator	W303-1B	Deletion	Sensitive	= Clonogenic survival<br>↑ PS exposure<br>↑ DNA fragmentation<br>↑ ROS accumulation	Laera et al., 2016
CDC35	Regulation of metabolism through cAMP generation	*C. albicans* SC5314	Deletion	Resistant	↑ Clonogenic survival	Phillips et al., 2006
COX20	Cox2p assembly into complex IV	BY4741	Deletion	Sensitive	↓ Clonogenic survival<br>↓ Metabolic activity	Kumar et al., 2015
CTT1	Catalase T	W303-1B	Overexpression	Resistant	↑ Clonogenic survival	Guaragnella et al., 2008
CYC1	Cytochrome *c*, isoform 1	W303-1B	W65S substitution	Resistant	↑ Clonogenic survival<br>↓ DNA fragmentation<br>↓ Cytochrome *c* release<br>↓ ROS accumulation<br>↑ Caspase-like activity	Guaragnella et al., 2011
CYC3	Heme lyase	W303-1A	Deletion	Resistant	↑ Clonogenic survival	Ludovico et al., 2002
DNM1	Mitochondrial fission factor	BY4741	Deletion	Resistant	↑ Clonogenic survival<br>↑ Metabolic activity	Fannjiang et al., 2004
					↓ Clonogenic survival	Teng & Hardwick, 2009
DOM34	Ribosomal subunit dissociation	BY4741	Deletion	Sensitive	↓ Clonogenic survival	Samanfar et al., 2017
EDC3	Enhancer of mRNA decapping	*C. albicans* BWP17	Double negative mutant	Resistant	↑ Clonogenic survival	Jung et al., 2014
EPL1	Histone modification	W303-1B	Overexpression	Sensitive	↑ PS exposure	Dong et al., 2017
ESA1	Histone modification	W303-1B	Overexpression	Sensitive	↑ PS exposure	Dong et al., 2017
FIS1	Mitochondrial fission factor	BY4741	Deletion	Sensitive	↓ Clonogenic survival<br>↓ Metabolic activity<br>↑ PS exposure<br>↑ Chromatin condensation	Fannjiang et al., 2004
					↓ Clonogenic survival	Teng & Hardwick, 2009
					↑ Chromatin condensation<br>↑ PS exposure<br>↑ DNA fragmentation	Sj et al., 2019

FKS1	Catalytic subunit of 1,3-β-D-glucan synthase	BY4741	Deletion	Sensitive	↓ Clonogenic survival	Mollapour, et al., 2009
FPS1	Plasma membrane aquaglyceroporin	BY4741	Deletion	Resistant	↑ Clonogenic survival	Mollapour & Piper, 2007
FUS3	MAPK involved in mating	BY4741	Deletion	Resistant	↑ Clonogenic survival<br>↓ Loss of PM integrity<br>↓ ROS accumulation	Rego et al., 2014
GCN2	Phosphorylation of the α-subunit of translation initiation factor	BY4742	Deletion	Resistant	↑ Clonogenic survival	Almeida et al., 2009
					↑ Clonogenic survival	Silva et al., 2013
GCN4	Transcriptional activator for amino acid biosynthesis	BY4742	Deletion	Resistant	↑ Clonogenic survival	Almeida et al., 2009
GIS1	Histone demethylase and transcription factor	BY4742	Deletion	Sensitive	↓ Clonogenic survival	Burtner et al., 2009
GUP1	Plasma membrane-bound *O*-acyltransferase	BY4741	Deletion	Sensitive	↓ Clonogenic survival<br>↑ Loss of PM integrity	Tulha & Lucas, 2018
		W303-1A	Deletion	Sensitive	↓ Clonogenic survival<br>↑ Chromatin condensation<br>= $\Delta\Psi_m$<br>↑ Loss of PM integrity<br>↑ ROS accumulation	Tulha et al., 2012
HAC1	Transcriptional activator involved in UPR	BY4742	Deletion	Sensitive	↓ Clonogenic survival	Kawazoe et al., 2017
HOG1	Mitogen-activated protein kinase involved in osmoregulation	BY471	Deletion	Sensitive	↓ Clonogenic survival	Mollapour & Piper, 2007
				Sensitive	↓ Clonogenic survival	Mollapour, Shepherd & Piper, 2009
				Unaffected	= Clonogenic survival<br>= Loss of PM integrity<br>= ROS accumulation	Rego et al., 2014
		W303-1B	Deletion	Sensitive	↓ Clonogenic survival<br>↑ DNA fragmentation<br>↑ ROS accumulation	Guaragnella et al., 2019
HOS1	Histone modification	BY4742	Deletion	Sensitive	↑ PS exposure	Dong et al., 2017
HPA2	Histone modification	W303-1B	Overexpression	Resistant	↓ PS exposure	Dong et al., 2017
HSC82	Cytoplasmic chaperone	BY4741	Deletion	Sensitive	↓ Clonogenic survival	Samanfar et al., 2017
		BY4742	Deletion	Sensitive	↓ Clonogenic survival<br>↑ Loss of PM integrity<br>↓ DNA fragmentation	Silva et al., 2013

HSP82	Cytoplasmic chaperone	BY4742	Deletion	Resistant	↑ Clonogenic survival<br>= Loss of PM integrity<br>↓ DNA fragmentation	Silva et al., 2013
HXK2	Phosphorylation of hexoses to hexose 6-phosphate	W303-1A	Deletion	Sensitive	↓ Clonogenic survival<br>↑ PS exposure<br>↑ ROS accumulation<br>↑ $\Delta\Psi_m$<br>↑ Mitochondrial fragmentation	Amigoni et al., 2013
					↓ Clonogenic survival<br>↑ PS exposure<br>↑ ROS accumulation	Amigoni et al., 2016
		W303-1B	Deletion	Sensitive	= Clonogenic survival<br>= DNA fragmentation	Laera et al., 2016
IRE1	Sensing unfolded proteins in the ER	BY4742	Deletion	Sensitive	↓ Clonogenic survival	Kawazoe et al., 2017
ISC1	Hydrolysis of sphingolipids to produce ceramide	BY4741	Deletion	Resistant	↑ Clonogenic survival<br>= Loss of PM integrity<br>↓ Cytochrome *c* release	Rego et al., 2020
		CG379	Deletion	Resistant	↑ Clonogenic survival<br>↓ ROS accumulation<br>↓ Mitochondrial degradation<br>↓ Cytochrome *c* release	Rego et al., 2012
KEX1	Cell death protease	SS328	Deletion	Resistant	↑ Clonogenic survival<br>↓ ROS accumulation	Hauptmann & Lhele, 2008
LAC1	Ceramide synthase component	CG379	Deletion	Resistant	↑ Clonogenic survival<br>↓ ROS accumulation<br>↓ Mitochondrial degradation<br>↓ Cytochrome *c* release	Rego et al., 2012
MDM10	ERMES complex component	BY4741	Deletion	Resistant	↑ Clonogenic survival<br>↓ Loss of PM integrity<br>↓ $\Delta\Psi_m$<br>↓ Mitochondrial degradation<br>↓ ROS accumulation<br>↓ Cytochrome *c* release	Martins et al., 2019
MDM12	ERMES complex component	BY4741	Deletion	Resistant	↑ Clonogenic survival<br>↓ Loss of PM integrity<br>↓ $\Delta\Psi_m$<br>↓ Mitochondrial degradation<br>↓ ROS accumulation<br>= Cytochrome *c* release	Martins et al., 2019

MDM34	ERMES complex component	BY4741	Deletion	Resistant	↑ Clonogenic survival<br>↓ Loss of PM integrity<br>↓ $\Delta\Psi_m$<br>↓ Mitochondrial degradation<br>↓ ROS accumulation<br>↓ Cytochrome *c* release	Martins et al., 2019
MDV1	Mitochondrial fission factor	BY4741	Deletion	Resistant	↑ Clonogenic survival<br>↑ Metabolic activity	Fannjiang et al., 2004
MID2	O-glycosylated plasma membrane protein	BY4741	Deletion	Resistant	↑ Clonogenic survival<br>↓ Loss of PM integrity<br>↓ ROS accumulation	Rego et al., 2014
MKK1/2	MAPKK involved in the PKC signaling pathway	BY4741	Deletion	Resistant	↑ Clonogenic survival<br>↓ Loss of PM integrity<br>↓ ROS accumulation	Rego et al., 2014
MSB2	Mucin involved in various signaling pathways	BY4741	Deletion	Resistant	↑ Clonogenic survival<br>↓ Loss of PM integrity<br>= ROS accumulation	Rego et al., 2014
NMA111	Serine protease and molecular chaperone	BY4741	Deletion	Resistant	↑ Clonogenic survival	Sokolov et al., 2006
NEM1	Phosphatase required for nuclear growth	BY4741	Overexpression	Resistant	↑ Clonogenic survival	Palermo et al., 2015
PBS2	MAPKK of the HOG signaling pathway	BY4741	Deletion	Resistant	↑ Clonogenic survival<br>↓ Loss of PM integrity<br>↓ ROS accumulation	Rego et al., 2014
PCA1	*S. pombe* metacaspase	*S. pombe* E6666	Deletion	Resistant	↑ Clonogenic survival	Agus et al., 2020
PDR18	Putative transporter of the ATP-binding cassette family	BY4741	Deletion	Sensitive	↓ Clonogenic survival<br>↑ Loss of PM integrity	Godinho et al., 2018
PEP3	Class C vacuolar protein sorting complex	BY4741	Deletion	Sensitive	↓ Clonogenic survival<br>↑ Loss of PM integrity<br>↓ DNA fragmentation<br>↑ ROS accumulation	Schauer et al., 2009

PEP4	Vacuolar aspartyl protease (proteinase A)	BY4741	Deletion	Resistant	↑ Clonogenic survival	Sousa et al., 2013
			Deletion	Sensitive	↓ Clonogenic survival<br>↑ Chromatin condensation	Alugoju et al., 2018a
			Deletion	Sensitive	↓ Clonogenic survival<br>↑ Chromatin condensation<br>↑ PS exposure<br>↑ DNA fragmentation	Sj et al., 2019
		W303-1B	Deletion	Sensitive	↓ Clonogenic survival<br>↑ Chromatin condensation<br>= $\Delta\Psi_m$<br>↓ Loss of PM integrity<br>↓ Mitochondrial degradation	Pereira et al., 2010
			Overexpression	Resistant	↑ Clonogenic survival<br>↑ Mitochondrial degradation	Pereira et al., 2010
PEP5	Class C vacuolar protein sorting complex	BY4741	Deletion	Sensitive	↓ Clonogenic survival<br>↑ Loss of PM integrity<br>↑ ROS accumulation	Schauer et al., 2009
PEX6	AAA-peroxin that participates in the recycling of Pex5p	BY4741	Deletion	Sensitive	↓ Clonogenic survival<br>↑ PS exposure<br>↑ Loss of PM integrity<br>↑ ROS accumulation	Jungwirth et al., 2008
PKH1	Serine/threonine kinase involved in sphingolipid-mediated signaling	BY4741	Deletion	Resistant	↑ Clonogenic survival<br>= Loss of PM integrity<br>= ROS accumulation<br>↓ Cytochrome *c* release	Rego et al., 2020
PMA1	Plasma membrane ATPase	BY4741	Overexpression	Resistant	↑ Clonogenic survival<br>↓ Loss of PM integrity<br>↓ ROS accumulation	Lee et al., 2016
PPH21	Catalytic subunit of protein phosphatase 2A	BY4742	Deletion	Resistant	↑ Clonogenic survival	Almeida et al., 2009
PPH22	Catalytic subunit of protein phosphatase 2A	BY4742	Deletion	Resistant	↑ Clonogenic survival	Almeida et al., 2009
POR1	Mitochondrial voltage-dependent anion channel	BY4741	Deletion	Sensitive	↓ Clonogenic survival<br>↑ Loss of PM integrity	Tulha & Lucas, 2018
		W303-1B	Deletion	Sensitive	↑ Clonogenic survival<br>↑ Chromatin condensation<br>↑ Loss of PM integrity<br>↑ DNA fragmentation<br>= ROS accumulation<br>↑ Cytochrome *c* release	Pereira et al., 2007
					↓ Clonogenic survival<br>↑ Cytochrome *c* release	Trindade et al., 2016

RAS1	Regulation of both MAPK and cAMP signaling pathway	*C. albicans* SC5314	Deletion	Resistant	↑ Clonogenic survival	Phillips et al., 2006
RAS2	Regulates nitrogen starvation, sporulation, and filamentous growth	BY4742	Deletion	Resistant	↑ Clonogenic survival	Burtner et al., 2009
RIM15	Protein kinase involved in cell proliferation	BY4742	Deletion	Sensitive	↓ Clonogenic survival	Burtner et al., 2009
RLM1	Transcription factor involved in the PKC-mediated MAPK pathway	BY4741	Deletion	Resistant	↑ Clonogenic survival<br>↓ Loss of PM integrity<br>↓ Cytochrome *c* release	Rego et al., 2014
RPL36A	Ribosomal 60S subunit protein	BY4741	Deletion	Sensitive	↓ Clonogenic survival	Samanfar et al., 2017
RTG2	Transcriptional activator of the RTG and TOR pathways	W303-1B	Deletion	Sensitive	↓ Clonogenic survival<br>↑ Chromatin condensation<br>= Cytochrome *c* release	Guaragnella et al., 2013
					↓ Clonogenic survival<br>↑ PS exposure<br>↑ Loss of PM integrity<br>↑ DNA fragmentation<br>↑ ROS accumulation	Laera et al., 2016
					↓ Clonogenic survival<br>↑ Chromatin condensation<br>↑ ROS accumulation	Guaragnella et al., 2019
RTG3	Transcription factor for RTG and TOR pathways	W303-1B	Deletion	Sensitive	↓ Clonogenic survival<br>↑ DNA fragmentation	Guaragnella et al., 2013
SCH9	AGC family protein kinase	BY4741	Deletion	Resistant	↑ Clonogenic survival<br>= Loss of PM integrity	Rego et al., 2020
		BY4742	Deletion	Resistant	↑ Clonogenic survival	Burtner et al., 2009
		DBY746	Deletion	Resistant	↑ Clonogenic survival	Burtner et al., 2009
SET2	Histone modification	W303-1B	Overexpression	Sensitive	↑ PS exposure	Dong et al., 2017
SGF29	Histone modification	BY4742	Deletion	Sensitive	↑ PS exposure	Dong et al., 2017
SHO1	Transmembrane osmosensor for filamentous growth and HOG pathways	BY4741	Deletion	Resistant	↑ Clonogenic survival<br>↓ Loss of PM integrity<br>↓ ROS accumulation	Rego et al., 2014

SIR2	Conserved NAD$^+$ dependent histone deacetylase	CML128	Inactivating mutations	Resistant	↑ Clonogenic survival	Vall-llaura et al., 2019
SIT4	Ceramide-activated, type 2A-related serine-threonine phosphatase	BY4741	Deletion	Resistant	↑ Clonogenic survival<br>= Loss of PM integrity	Rego et al., 2020
		BY4742	Deletion	Resistant	↑ Clonogenic survival	Almeida et al., 2009
SLT2	Serine/threonine MAP kinase	BY4741	Deletion	Resistant	↑ Clonogenic survival<br>↓ Loss of PM integrity<br>↓ Cytochrome *c* release	Rego et al., 2014
SNF1	AMP-activated serine/threonine protein kinase	W303-1A	Deletion	Sensitive	↓ Clonogenic survival<br>↑ PS exposure<br>↑ ROS accumulation	Bonomelli et al., 2020
SOD1	Cytosolic copper-zinc superoxide dismutase	W303-1B	Overexpression	Sensitive	↓ Clonogenic survival	Guaragnella et al., 2008
SSK22	MAPKKK of HOG1 mitogen-activated signaling pathway	BY4741	Deletion	Resistant	↑ Clonogenic survival<br>↓ Loss of PM integrity<br>↓ ROS accumulation	Rego et al., 2014
STE2	Receptor for α-factor pheromone	BY4741	Deletion	Resistant	↑ Clonogenic survival<br>↓ Loss of PM integrity<br>↓ ROS accumulation<br>↓ Cytochrome *c* release	Rego et al., 2014
STE5	Pheromone-responsive MAPK scaffold protein	BY4741	Deletion	Resistant	↑ Clonogenic survival<br>↓ Loss of PM integrity<br>↓ ROS accumulation	Rego et al., 2014
STE11	Signal transducing MEK kinase	BY4741	Deletion	Resistant	↑ Clonogenic survival<br>↓ Loss of PM integrity<br>= ROS accumulation	Rego et al., 2014
TEL1	Telomere length regulation	BY4741	Deletion	Sensitive	↓ Clonogenic survival<br>↑ Chromatin condensation<br>↑ PS exposure<br>↑ Loss of PM integrity	Alugoju et al., 2018b
TOR1	PIK-related protein kinase involved in growth control	BY4741	Deletion	Resistant	↑ Clonogenic survival<br>↓ ROS accumulation	Büttner et al., 2011
		BY4742	Deletion	Resistant	↑ Clonogenic survival<br>↓ DNA fragmentation<br>↓ ROS accumulation	Almeida et al., 2009
VMA3	Subunit of vacuolar ATPase	BY4741	Deletion	Sensitive	↓ Clonogenic survival	Konarzewska et al., 2017
VPS16	Class C vacuolar protein sorting complex	BY4741	Deletion	Sensitive	↓ Clonogenic survival<br>↑ Loss of PM integrity<br>↑ ROS accumulation	Schauer et al., 2009

VPS33	Class C vacuolar protein sorting complex	BY4741	Deletion	Sensitive	↓ Clonogenic survival<br>↑ Loss of PM integrity<br>↑ ROS accumulation	Schauer et al., 2009
YAF9	Histone modification	BY4742	Deletion	Sensitive	↑ PS exposure	Dong et al., 2017
		W303-1B	Overexpression	Sensitive	↑ PS exposure	Dong et al., 2017
YCA1	Ca^{2+}-dependent cysteine protease	W303-1B	Deletion	Resistant	↑ Clonogenic survival<br>= Chromatin condensation<br>↓ Caspase-like activity	Guaragnella et al., 2006
					↑ Clonogenic survival<br>= Chromatin condensation<br>= ROS production<br>↓ Caspase-like activity<br>= Cytochrome *c* release	Guaragnella et al., 2010a
					↑ Clonogenic survival<br>= DNA fragmentation<br>↓ Caspase-like activity	Guaragnella et al., 2010b
					↑ Clonogenic survival	Antonacci et al., 2012
					↑ Clonogenic survival<br>= DNA fragmentation<br>= Mitochondrial fragmentation	Longo et al., 2015
YPK1	Serine/threonine kinase	BY4741	Deletion	Resistant	↑ Clonogenic survival<br>= Loss of PM integrity	Rego et al., 2020
YSP2	Sterol-binding protein	W303-1B	Deletion	Resistant	↑ Clonogenic survival	Sokolov et al., 2006
